# Resurrection of Nalidixic Acid: Evaluation of Water-Based Nanoformulations as Potential Nanomedicine

**DOI:** 10.1186/s11671-018-2718-8

**Published:** 2018-09-24

**Authors:** Alka Pandey, Nisha Aggarwal, Alok Adholeya, Mandira Kochar

**Affiliations:** 1 TERI-Deakin Nanobiotechnology Centre, TERI Gram, The Energy and Resources Institute, Gwal Pahari, Gurugram, Haryana 122003 India; 2 Department of Chemistry, Sri Aurobindo College, University of Delhi, New Delhi, India

**Keywords:** Nalidixic acid, Antibiotics, Hydrazine, Nanoformulation, Biosafety

## Abstract

**Abstract:**

Resistance to quinolone antibiotics has been a serious problem ever since nalidixic acid was introduced into clinical medicine. Over time, resistance of pathogenic microbes to nalidixic acid led to the design of novel variants to revive its potential application. In the present work, a series of eight nanoformulations of nalidixic acid-based diacyl and sulfonyl acyl hydrazine derivatives were prepared. All nanoformulations were found to be stable at different storage temperatures. Antibacterial and anticandida activity of the eight nanoformulations presented encouraging results when compared with their non-nano parent counterparts. The nanoformulations of chloro, furanyl, and sulfonyl acyl substituted derivatives of nalidixic acid displayed most promising results (MIC ranging from 50 to 100 μg mL^−1^) against the tested bacteria and yeast. Among the screened bacteria, *Acinetobacter baumannii* displayed maximum sensitivity to the above nanoformulations. Biosafety study on the mammalian model—wax moth, *Galleria mellonella*—showed that all eight prepared nanoformulations were absolutely nontoxic to the larvae and subsequent pupae and hence may likely have no or low toxicity against mammalian systems.

**Graphical Abstract:**

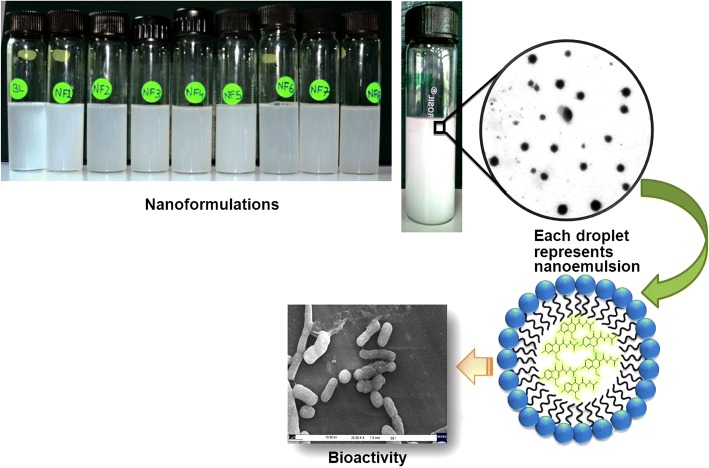

**Electronic supplementary material:**

The online version of this article (10.1186/s11671-018-2718-8) contains supplementary material, which is available to authorized users.

## Background

Antibiotics have revolutionized our capabilities to combat infectious diseases without undergoing intricate medical surgeries. Resistance to antibiotics is one of the greatest intimidations to global health leading to higher medical expenses and increased mortality rate [1–9]. Consequently, there is an urgent need for the development of new, more effective, and safe antibiotics. Quinolones have been the most frequently prescribed class of antibiotics used for the treatment of wide variety of infectious diseases in humans [10, 11]. However, over the years, the excessive use of quinolones resulted in development of resistance against a wide range of pathogenic Gram-positive and Gram-negative bacteria [12–16]. Nalidixic acid, the first synthetic quinolone, was primarily used for the treatment of urinary tract infection caused by Gram-negative bacteria. However; it is no longer in clinical use due to bacterial resistance and development of more effective antimicrobials. Several amendments were made recently on the basic quinolone nucleus to synthesize new 1,2,4-triazole, diacyl, and sulfonyl acyl hydrazine derivatives of nalidixic acid to improve antibacterial spectrum and pharmacokinetics properties of the parent molecule. Most of these hybrid molecules prepared by the amalgamation of different pharmacophores represented better antimicrobial profile than the parent compound [17, 18]. Such new antibacterial agents with appropriate molecular alterations and enhanced potency are further explored in the coming future to overcome antibiotic resistance.

Over the recent years, various nanotechnological approaches have been explored as a promising alternative in design, development, and delivery of new and more active antimicrobial drugs, precisely in overpowering antibiotic-resistant bacteria and increasing the efficiency of existing antibiotics [19–22]. The promising physiochemical behavior of “nano” sized materials including small and controllable size, large surface area to mass ratio, high stability, functionalizing potential, improved drug solubility, and protracted systemic bioavailability can be explored in combating antibiotic resistance. In the recent years, several nanoparticles and nanoparticle-modulated delivery vehicles or nano-transporters have received significant attention for the physical entrapment, adsorption, or chemical conjugation of active drug molecules for enhanced pharmacokinetic and therapeutic behavior [23–25]. In the quest for the development of more effective and safe antibiotics, we envisioned to design and formulate innovative, water-based nanoformulations of diacyl and sulfonyl acyl hydrazine derivatives of nalidixic acid. All newly prepared nanoformulations of nalidixic acid hydrazine derivatives were tested in vitro for their antibacterial potential as well as against yeast and mammalian model system.

## Methods/Experimental

All chemicals and solvents were purchased from Merck and used as such without further purification. The oils used were locally purchased. Culture growth media and its components were purchased from Hi Media (Mumbai, India). The progress of the reaction was monitored by thin layer chromatography (TLC) using pre-coated Merck silica gel 60 F254 TLC plates. The spots were visualized under ultraviolet light (UV) or by iodine vapor. Infra-red (IR) spectra were recorded using a Nicolet 6700 FT-IR (Thermo Fischer Scientific, USA) spectrophotometer as KBr pellets and expressed as *ν*_max_ in cm^−1^. The characterization of synthesized compounds was done by ^1^H NMR and ^13^C NMR spectroscopy on BrukerAvanceII400 spectrometer at 400 and 100 MHz, respectively in CDCl_3_ using tetramethylsilane (TMS) as an internal standard. The absorption spectra were recorded at room temperature by UV–Visible absorption spectrophotometer (UV-2450, Shimadzu, Japan).

### Synthesis of Diacyl and Sulphonyl Acyl Hydrazine Derivatives of Nalidixic Acid

Nalidixic acid diacyl hydrazine and sulfonyl acyl hydrazine derivatives were synthesized as described previously (see Additional file 1: Figure S1) [18]. The products obtained (1–6 (diacyl hydrazine derivatives) and 7–8 (sulfonyl acyl hydrazine derivatives)**,** respectively) were used for nanoformulation preparation.

### Preparation of Nanoformulations

Water-based nanoemulsions of nalidixic acid hydrazine derivatives were prepared using various oils, surfactants, and water. All the components used in the formulations were pharmaceutically suitable, non-irritant, and under the category of generally regarded as safe (GRAS) by Food and Drug Administration [26]. Ultrasonic disintegration method [27] was used for preparing oil in water nanoemulsion by employing different combinations of sesame oil, olive oil, corn oil, pine oil, cedarwood oil, soyabean oil, and β-citronellol and surfactants like tween 20, tween 80, triton-x-100, and PF68. The ratio of active drug, oil, surfactant, and water for the preparation of stable and clear nanoemulsion were optimized. Coarse emulsions were initially prepared by uniformly mixing selected oils containing active ingredient dissolved in 1 mL of ethanol and surfactants in different ratios 1:0.5, 1:0.75, 1:1, 1:1.5 1:2, 1:2.5, 1:3. 1:3.5, 1:4, 1:4.5, and 1:5 (*w*/*w*) followed by the slow addition of water (Milli-Q, Millipore) with constant stirring, to formulate stable emulsions. The results showed that 1:3 ratio of oil (olive oil)-surfactant (tween 80) was most stable under ambient conditions. The microemulsions were then sonicated with 20 kHz sonicator probe (Ultrasonics, USA), immersed directly into the solution, with a power output of 750 W. The conditions for sonication were constantly set as follows: energy: 20 kHz, pulser 5 s, amplitude 30%, temperature 4 °C, time maximum of 1 h. The sample which was found to be stable for more than 30 days was taken for further characterization.

### Characterization of Nanoformulations

Droplet size and dispersity index (DI) of all eight nanoformulations was measured by dynamic light scattering (DLS) technique (ZS90, Malvern, UK) in triplicate as an average of 15 to 20 runs per measurement at 25 °C. Each sample was diluted ten times with water (Milli-Q, Millipore) before measurement. The elapsed time was set to be 6 min to achieve better fitting results. Droplet size was measured in nanometers (nm). The average hydrodynamic radius was calculated by the program based on Stokes-Einstein equation.

Furthermore, structure and morphology of nanoemulsions was studied by transmission electron microscopy (TEM). A drop of nanoemulsion (10 μL) dispersed in water (Milli-Q, Millipore) was directly dispersed on shiny copper grid placed on a filter paper (Whatman No. 1). After drying at room temperature for 1 h, samples were taken for TEM imaging (Tecnai G^2^ T20, TWIN, Electron source: Lab6 filament, 60–200 kV). A combination of bright field imaging at increasing magnification and off diffraction mode was used to determine the shape and size of the nanoemulsions.

All eight nanoformulations were stored at controlled laboratory conditions and monitored regularly for any change in their droplet size, DI, pH, and conductivity as a function of time. Stability of nanoformulations was checked by DLS techniques and visual observation for any change in their form, flocculation, aggregation, or precipitation. Thermal stability was also checked at different storage temperatures (4 °C and 25 °C). Also, the nanoformulations were subjected to centrifugation at 3000 rpm for 15 min and checked for any visual phase separation. The stability of active ingredient entrapped in nanoemulsions was determined by UV–Vis absorption spectrophotometry by centrifuging nanoemulsions (1 mL) at 15,000 rpm for 30 min. The supernatant was diluted with water (1 mL) and absorption was measured in triplicate at 257, 205, 246, 205, 254, 256, 257, and 208 nm for nanoformulations NF1, NF2, NF3, NF4, NF5, NF6, NF7, and NF8, respectively. The amount of active ingredient entrapped in the supernatant was calculated by the following relationship [28]

Entrapment efficiency (%)  =  total AI – free AI/total AI  ×  100

### In Vitro Antimicrobial Activity

The antimicrobial activity of nanoformulations was assessed against four bacterial strains including *Staphylococcus aureus* (MTCC 11949), *Bacillus subtilis* (NEUB), *Pseudomonas aeruginosa* (NRRL B59191), and *Acinetobacter baumannii* (MTCC 9869) and *Candida albicans* (SC5314). The bacterial and yeast stock cultures were incubated for 24 h at 30 °C on nutrient agar and yeast peptone dextrose agar (YPDA) medium (Hi Media, Mumbai, India), respectively.

A loopful of bacteria and yeast was taken from the pure culture and inoculated into 10 mL of nutrient broth and yeast peptone dextrose broth (Hi media, Mumbai, India), respectively. The broth suspension was then incubated at 150 rpm, 30 °C for 4 h. The growth obtained was used as inoculum for the sensitivity assay. Antibacterial activity of the prepared nanoformulations was carried out at concentrations 800, 400, 200, 100, 50 μg mL^−1^ along with non-nano derivatives and tetracycline as standard antibiotic by broth microdilution method against all test agents as per the Clinical and Laboratory Standards Institute (CLSI) guidelines [29] in 96-well microplates using MTT (3-(4,5-dimethyl-2-thiazolyl)-2,5-diphenyl-2H-tetrazolium bromide) dye. Fresh cultures of bacteria *S*. *aureus*, *B*. *subtilis*, *P*. *aeruginosa*, and *A*. *baumannii* was taken from log phase cultures and diluted with nutrient broth to set 0.5 OD (as per McFarland standard, 9.8 × 10^8^ CFU/mL for *S*. *aureus*, 5.8 × 10^7^ CFU/mL for *B*. *subtilis*, 8.7 × 10^7^ CFU/mL for *P*. *aeruginosa*, and 1.23 × 10^8^ CFU/mL for *A*. *baumannii*). Twenty microliters of this bacterial inoculum was then added to each well containing different concentration of the nanoformulations. All tests were performed in triplicate. The plate was then incubated for 18 h at 30 °C. Tetracycline was used as positive control and nutrient broth as negative control. After incubation, change in OD was recorded at 570 nm using a microplate reader and compared with that of the initial OD. Also, total number of viable cells present in each treatment was confirmed by CFU analysis after appropriate serial dilution. After this, 10 μL of MTT dye (5 mg mL^−1^) was added in each well and further incubated for 4 h. The microtiter plate with bacteria was then examined for color change. Hundred microliter of DMSO was added to each well after careful removal of bacterial cells. Wells with viable bacterial cells showed change in color from yellow to purple. Absorbance was finally recorded using a microplate reader at 570 nm.

The mode of antibacterial activity was studied using scanning electron microscopy (SEM); fresh cultures of *S. aureus* and *A*. *baumannii* were treated with 800, 400, and 200 μg mL^−1^ of the nanoformulations, NF7 and NF4 respectively. Tetracycline and parent nalidixic acid at 200 μg mL^−1^ concentrations were used as standards and sterile water as control. Treated cells after 18 h of nanoformulation exposure were fixed in 2.5% (*v*/*v*) glutaraldehyde solution buffered with 0.1 M phosphate buffer (pH 7.2) overnight at 4 °C. After fixation, cells were washed three times in MilliQ water followed by dehydration through a graded ethanol series (10–100%). The treated samples were mounted on aluminum stubs, sputter-coated with gold-palladium (QuorumTechnologies SC7620, Berkshire, UK), and examined under a scanning electron microscope (EVO MA10, Carl Zeiss).

Anticandida activity was performed by agar well diffusion method on YPDA artificial media containing yeast extract powder (10%), peptone (20%), glucose (20%), and purified agar powder (20%) [30]. The inoculum was prepared using 24-h plate cultures of *Candida albicans*. The colonies were suspended in 0.85% saline and the turbidity was compared with the 0.5 McFarland standard, to produce a yeast suspension of 1.5 × 10^8^ CFU/mL. Fifty milliliters of YPDA media was autoclaved and cooled to 35–40 °C and then aseptically inoculated with 1 mL of inoculum suspension (calibrated to 0.5 McFarland standard). The inoculated media was poured into the assay plate (150 cm in diameter) and allowed to cool down on a leveled surface. Once solidified, six wells, 4 mm in diameter, were cut out of the agar, and 50 μL of the nanoformulations at 800, 400, 200, 100, and 50 μg mL^−1^ concentrations were carefully placed into each well. All experiments were carried out in triplicate. The plates were incubated at 37 °C for 18–24 h. Cycloheximide and Nystatin were used as positive controls. Anticandida activity was determined by measuring the zone of inhibition. The lowest concentration at which a clear zone was formed was considered as the minimum inhibitory concentration (MIC).

### Biosafety Assay on *Galleria mellonella* Larvae

Wax moth, *Galleria mellonella* (Lepidoptera: Pyralidae) larvae are increasingly being explored as mammalian model system to study microbial infections and pathogenesis of many bacteria and fungi [31–33]. *G*. *mellonella* larvae can be easily and economically obtained and reared in large numbers under normal lab conditions as compared with other classical mammalian model hosts. Their distinctive immune responses demonstrate significant resemblances with the immune response in vertebrates and show both cellular and humoral defenses and thus are good first line model system. Also, there are no ethical restrictions and their short life cycle makes them perfect model for large-scale studies [34, 35].

Eggs of *Galleria mellonella* were obtained from Division of Nematology, Indian Agricultural Research Institute, New Delhi, India and reared in the lab for successive generations on an artificial diet [36] at 28° ± 2 °C and 60 ± 5% relative humidity in dark. The adult moths were fed with 10% honey solution in mating jars. Tissue paper folded in a fan shape was provided for egg laying in the mating jars. The eggs were seeded in the freshly made artificial diet and the newly hatched insect larvae were allowed to develop. The final fifth instar larvae (2–3 cm long, creamy colored, and 180–250 mg in weight), which develop from the egg after about 5 weeks, were used for biosafety experiment of nanoformulations. Biosafety assay was performed by direct injection and spray method. The larvae were stored at 15 °C before use and starved for 24 h before injection of test nanoformulation. A group of ten healthy larvae of *G*. *mellonella* approximately similar in weight was selected for each treatment. In direct injection method, each larva was injected with 10 μL of 1000, 800, 400, and 200 μg mL^−1^ concentration of prepared nanoformulations along with blank nanoformulation in the left posterior proleg using Dispo Van superfine short needle syringe (31G × 5/16, 0.25 × 8 mm). The syringes were changed between treatments and with different concentrations. Cypermethrin 25% EC was used as positive control while sterile water acted as negative control. All tests were performed in duplicate. After treatment, the larvae were incubated in petri plates at 28° ± 2 °C and survival was monitored regularly after 24, 48, and 72 h. In case of spray method, each larva was uniformly sprayed with the active ingredient. After treatment with appropriate dose of nanoformulation, larvae were incubated at 28° ± 2 °C and survival was monitored regularly after 24, 48, and 72 h. The larvae were considered dead when they did not respond to physical stimulation (gentle pressure with forceps).

## Results and Discussion

The extensive use of antibiotics has led to many threats to public wellbeing including deadly multidrug resistance. In the search for the development of novel and more effective antibiotics, we have explored the immeasurable potential of nanotechnology for the preparation of water-based, highly effective, and ready to use nanoformulations of substituted, diacyl hydrazine, and sulfonyl acyl hydrazine derivatives of the antibiotic, nalidixic acid. The synthesis of the active compounds was achieved by method previously reported in the literature (see Additional file 1: Figure S2) [18]. Six nalidixic acid diacyl hydrazine derivatives, namely N′-(2-chlorobenzoyl)-1-ethyl-7-methyl-4-oxo-1,4-dihydro-1,8-naphthyridine-3-carbo hydrazide (1), N′-(3-chlorobenzoyl)-1-ethyl-7-methyl-4-oxo-1,4-dihydro-1,8-naphthyridine-3-carbo hydrazide (2), N′-(4-chlorobenzoyl)-1-ethyl-7-methyl-4-oxo-1,4-dihydro-1,8-naphthyridine-3-carbo hydrazide (3), N′-(3-bromobenzoyl)-1-ethyl-7-methyl-4-oxo-1,4-dihydro-1,8-naphthyridine-3-carbo hydrazide (4), N′-(4-bromobenzoyl)-1-ethyl-7-methyl-4-oxo-1,4-dihydro-1,8-naphthyridine-3-carbo hydrazide(5), and 1-Ethyl-N′-(furan-2-carbonyl)-7-methyl-oxo-1,4-dihydro-1,8-naphthyridine-3-carbo hydrazide (6), and two nalidixic acid sulfonyl acyl hydrazine derivatives, 4-chloro-N′-(1-ethyl-7-methyl-4-oxo-1,4-dihydro-1,8-naphthyridine-3-carbonyl)-benzene sulphonohydrazide (7) and N′-(1-ethyl-7-methyl-4-oxo-1,4-dihydro-1,8-naphthyridine-3-carbonyl)-4-nitrobenzene sulphonohydrazide (8), were synthesized. The structure of all synthesized compounds were confirmed by their IR, ^1^H, and ^13^C NMR spectra and were found concordant with the reported values [18].

**Fig. 1:**
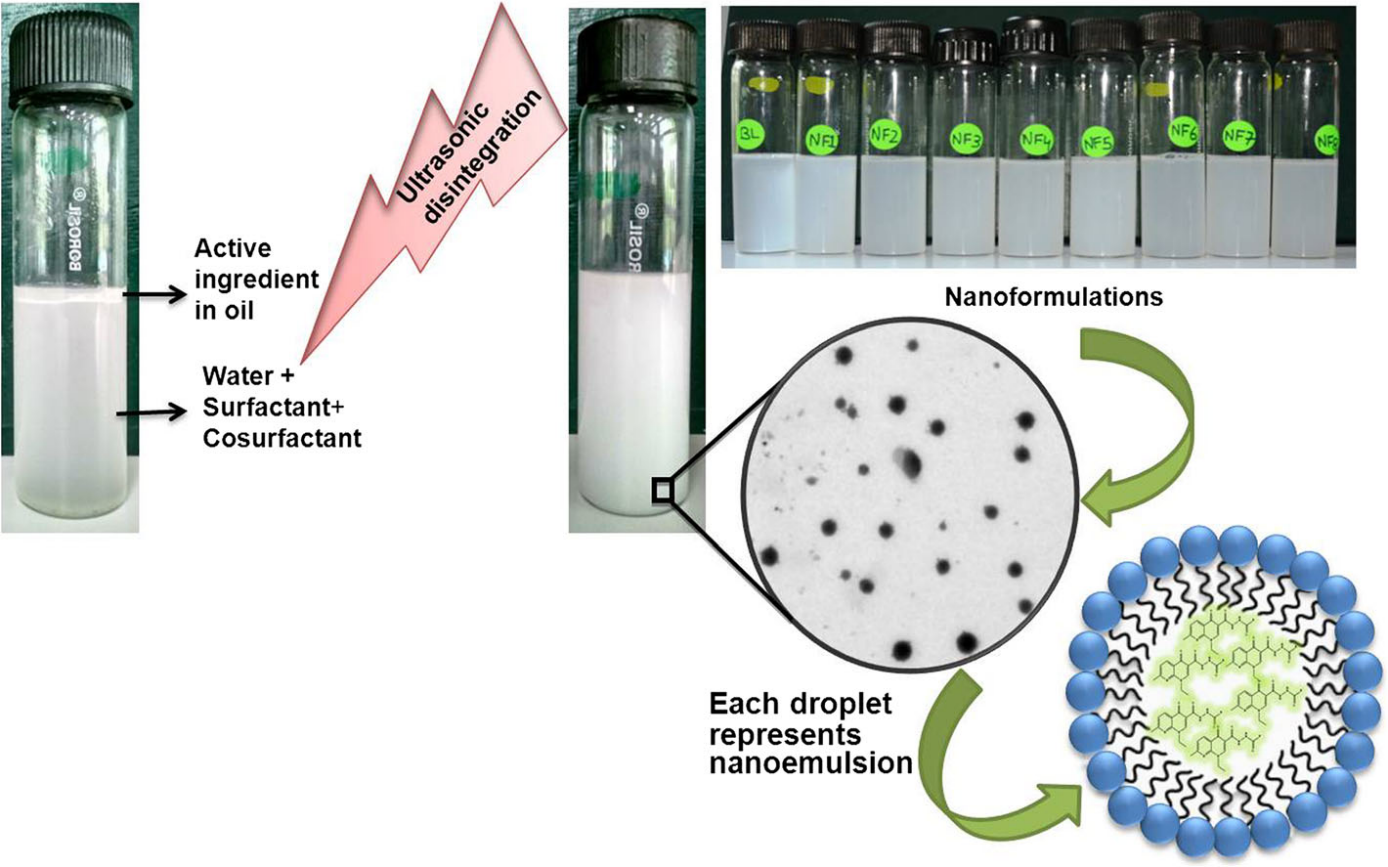
Method of nanoformulation preparation

**Fig. 2:**
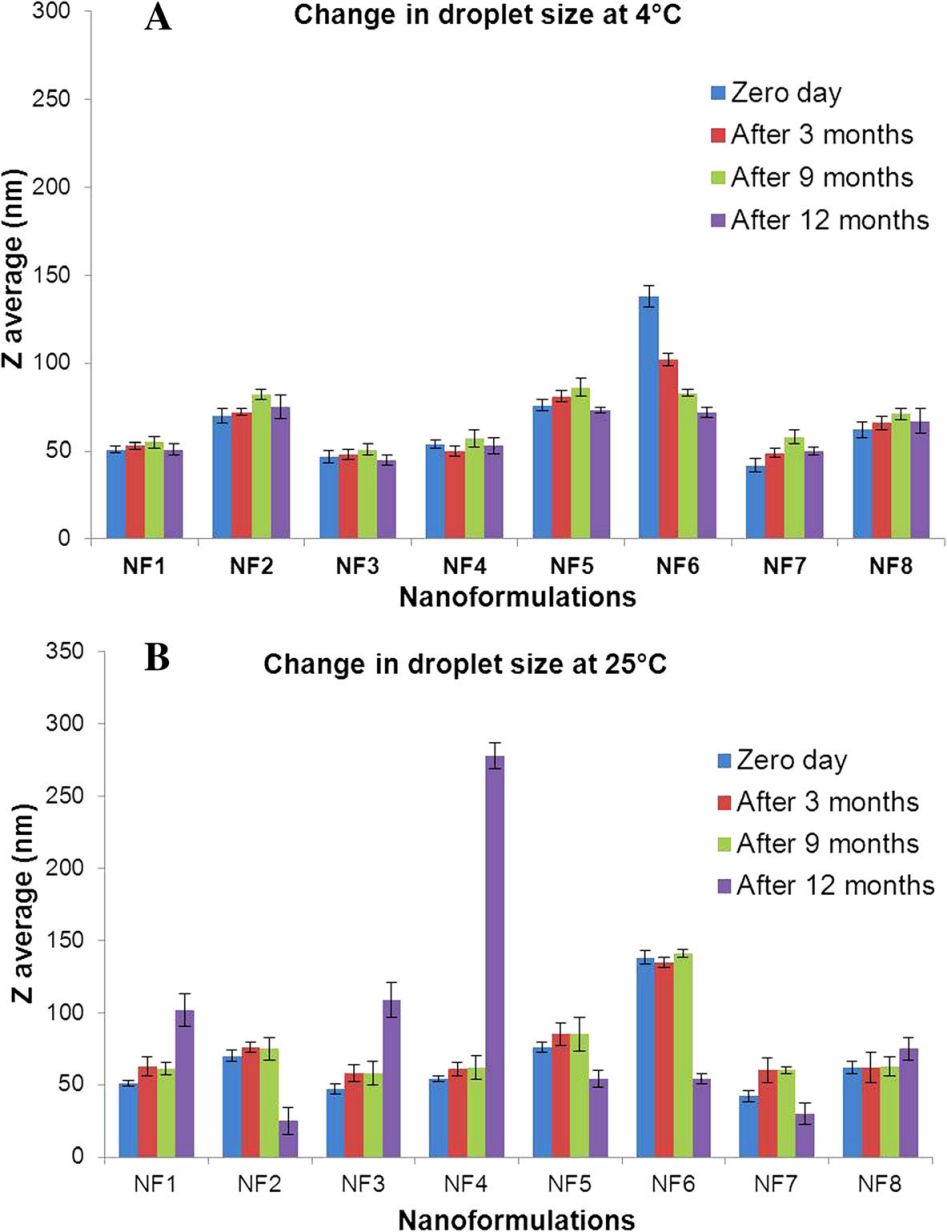
Stability of prepared nanoformulations. **a** At temperature 25 °C. **b** At temperature 4 °C (error bars represents standard deviation)

**Fig. 3:**
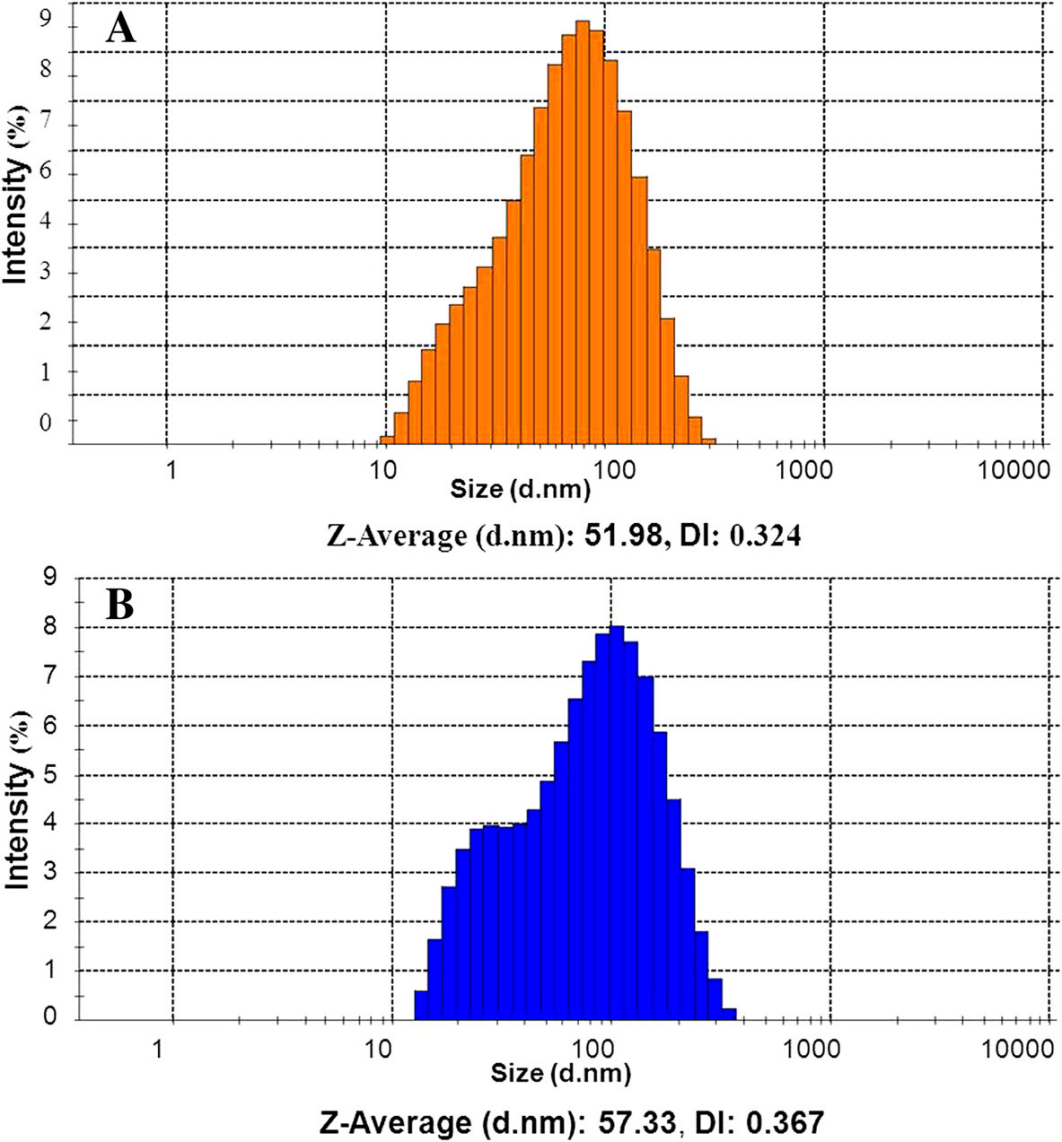
Particle size distribution of nanoformulation, NF1. **a** Untreated nanoformulation. **b** Nanoformulation exposed to UV radiation (50 Hz) for 48 h

**Fig. 4:**
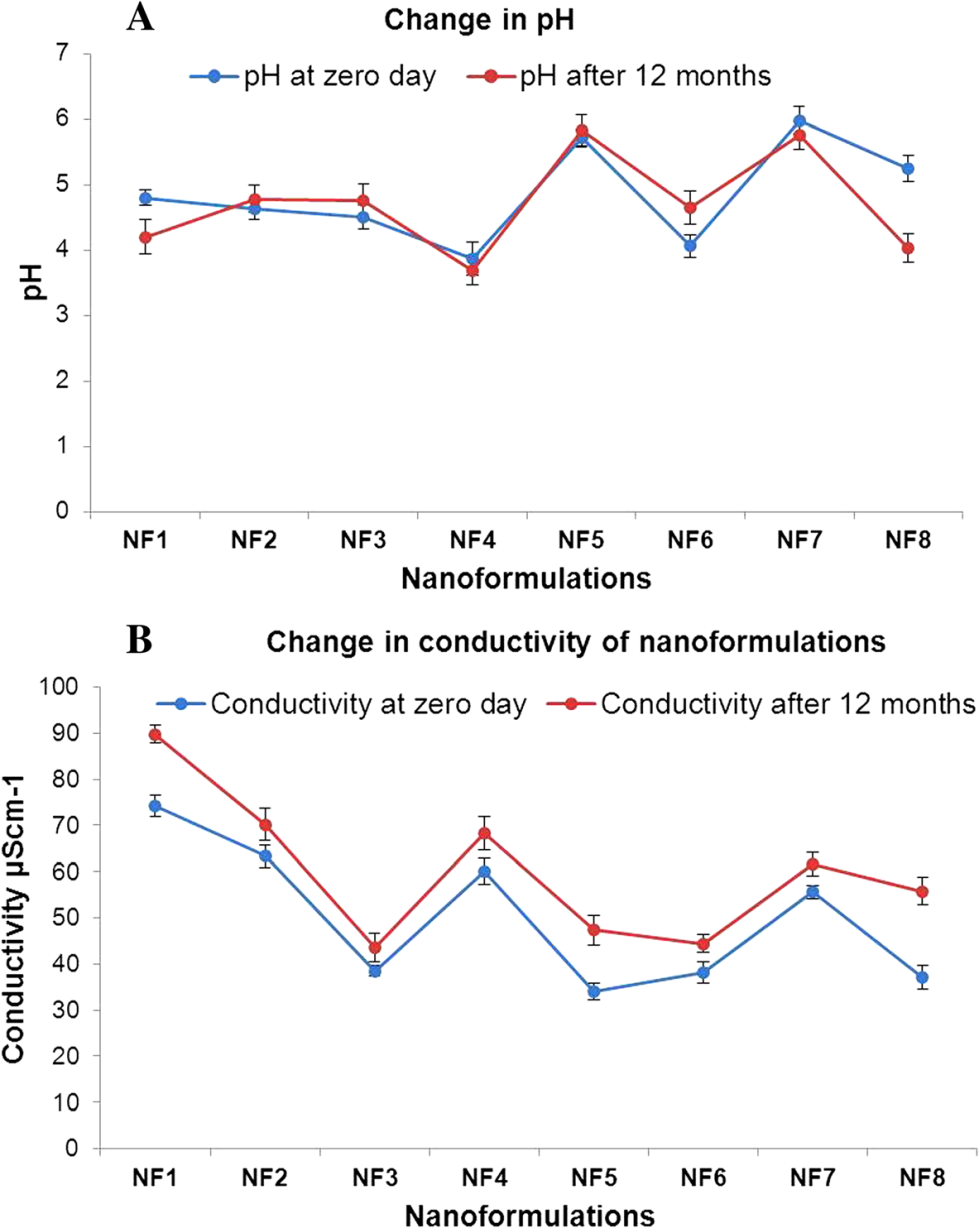
Change in **a** conductivity and **b** pH of nanoformulations with time (error bars represents standard deviation)

Oil in water nanoformulations of nalidixic acid derivatives were prepared by employing high-energy ultrasonic disintegration method (Fig. 1) in a manner similar to that reported [37]. The stability of all prepared nanoformulations was tested over a function of time and maximum stability was observed in combination of olive oil and tween 80 in 1:3 ratio. The physiochemical behavior and the average droplet size of all prepared nanoformulations were presented in Table 1. The mean droplet size of nanoemulsion was found to be in the range 42–138 nm. The average droplet size of nanoformulations NF7 and NF3 was less than 50 nm while nanoformulations NF1, NF2, NF4, NF5, and NF8 had average droplet size between 51 and 70 nm, respectively. NF6 had largest droplet size of 138 nm. The low DI values in almost all nanoformulations indicated monodisperse behavior of droplets in the formulation. The size distribution of each nanoformulation has been depicted in Additional file 1: Figure S2. DLS results showed no significant change in the diameter of nanoemulsions even after more than 12 months of storage at different temperature conditions. However, in the case of nanoformulation, NF6 gradual disintegration of nanoemulsions into smaller droplets was seen when stored at 4 °C as depicted in Fig. 2a. The change in average droplet size distribution and DI at 4 ± 2 °C is given in Additional file 1: Table S1a. Conversely, the stability results at 25 ± 2 °C illustrate that all nanoformulations were stable for 9 months as shown in Fig. 2b. After 9 months of storage, aggregation of smaller of droplets into larger nanoemulsions was seen in case nanoformulations NF1, NF3, and NF4. However, for nanoformulations NF2, NF5, NF6, and NF7, disintegration of droplets was seen as shown in Fig. 2a, b. The droplet size of NF8 remained almost constant even after 12 months of storage. The change in average droplet size distribution and DI at 25 ± 2 °C is given in Additional file 1: Table S1b. Above results confirmed that all nanoformulations except NF6 were stable at 4 ± 2 °C and nanoformulation NF8 possesses maximum stability after 12 months of storage at 25 ± 2 °C. Apart from storage time, these nanoformulations were also found to be stable when exposed to UV irradiation (50 Hz) for 48 h. The average droplet size distribution and DI of representative nanoformulation (NF1) is shown in Fig. 3. All prepared nanoformulations were kinetically stable systems with no phase separation, creaming, or coalescence at different temperatures (4 ± 2 °C and 25 ± 2 °C). Also, all nanoemulsions were found stable without any phase separation after centrifugation at 3000 rpm for 15 min. Moreover, no significant change in pH and conductivity of nanoformulation was seen with time as depicted in Fig. 4a, b respectively.

**Table 1:** Physicochemical properties of nanoformulations

S.No.	Z average (nm)	Dispersity index (DI)	pH	Conductivity (μS cm^−1^)	Percentage entrapment efficiency (%)
NF1	51 ± 2.08	0.324	4.80 ± 0.11	74.3 ± 2.27	85 ± 2.01
NF2	70 ± 4.04	0.337	4.62 ± 0.16	63.4 ± 2.43	91 ± 1.63
NF3	47 ± 3.60	0.284	4.50 ± 0.18	38.5 ± 1.15	83 ± 1.35
NF4	54 ± 2.08	0.226	3.87 ± 0.25	60.1 ± 2.81	86 ± 2.08
NF5	76 ± 3.21	0.194	5.72 ± 0.13	34 ± 1.71	82 ± 2.13
NF6	138 ± 6.02	0.288	4.06 ± 0.16	38.1 ± 2.27	81 ± 1.94
NF7	42 ± 3.60	0.339	5.98 ± 0.20	55.6 ± 1.50	88 ± 2.01
NF8	62 ± 4.50	0.396	5.25 ± 0.19	37.1 ± 2.48	81 ± 1.13

Z average, pH, conductivity, and % data are represented with ± standard deviation

Nanoemulsions prepared from vegetable oils generally display lower pH values; this is mainly attributed to the fact that during nanotization, the fatty acid esters present in vegetable oils gets hydrolyzed into free fatty acids [38]. The prepared nanoemulsions in the present study displayed stable pH values after 12 months when kept at different temperatures of 4 ± 2 °C and 25 ± 2 °C. Like pH, change in electrical conductivity also has substantial influence on nanoemulsion stability [38]. The electrical conductivity of the nanoformulation showed very slight difference over a period of time at different temperature conditions, therefore not significantly affecting overall droplet size. All oil-in-water nanoemulsions presented here displayed high physical stability over a period of time with constant pH, electrical conductivity, and stable droplet size at different temperatures.

**Fig. 5:**
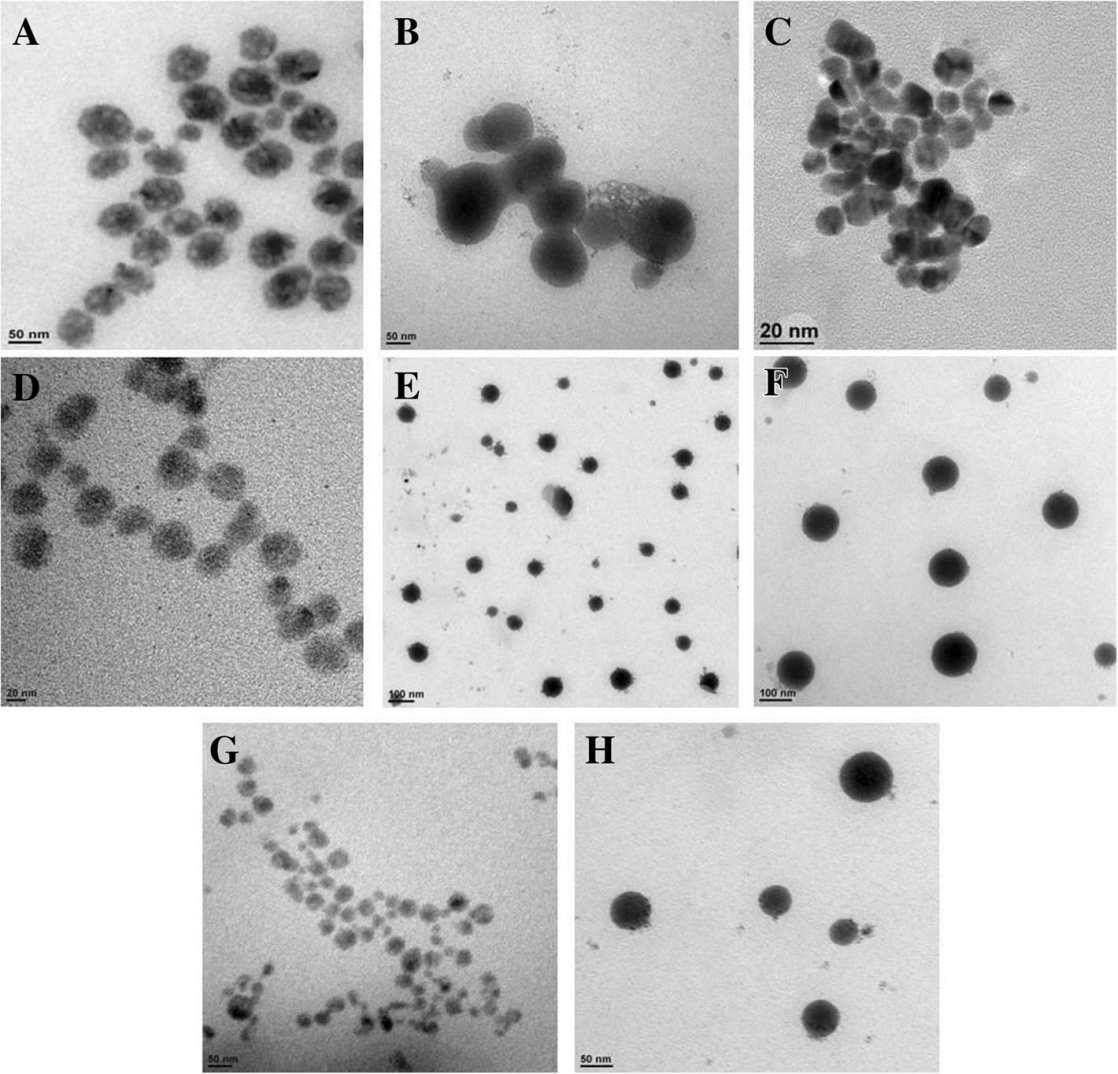
TEM images of prepared nanoformulations. **a** NF1. **b** NF 2. **c** NF3. **d** NF4. **e** NF5. **f** NF6. **g** NF7. **h** NF8 at 0 day (scale bar is shown at the bottom left corner of each figure and varied between 20 and 100 nm)

The morphology of nanoformulations was studied by TEM. The shape of the nano droplets was found to be spherical (Fig. 5) and the particle size of the emulsion droplets was found to be in the range of nanometers. These particles were randomly dispersed and distributed throughout the field as observed under TEM. Though, aggregation was not seen, in addition, joint particles appeared in the picture. This could be credited to the small diameter of nanoemulsions. Change in morphology of nanoemulsions over a period of time was also examined by TEM. Results showed that the shape and size of nanoemulsions remained more or less similar even after 3 months of storage (see Additional file 1: Figure S3).

**Fig. 6:**
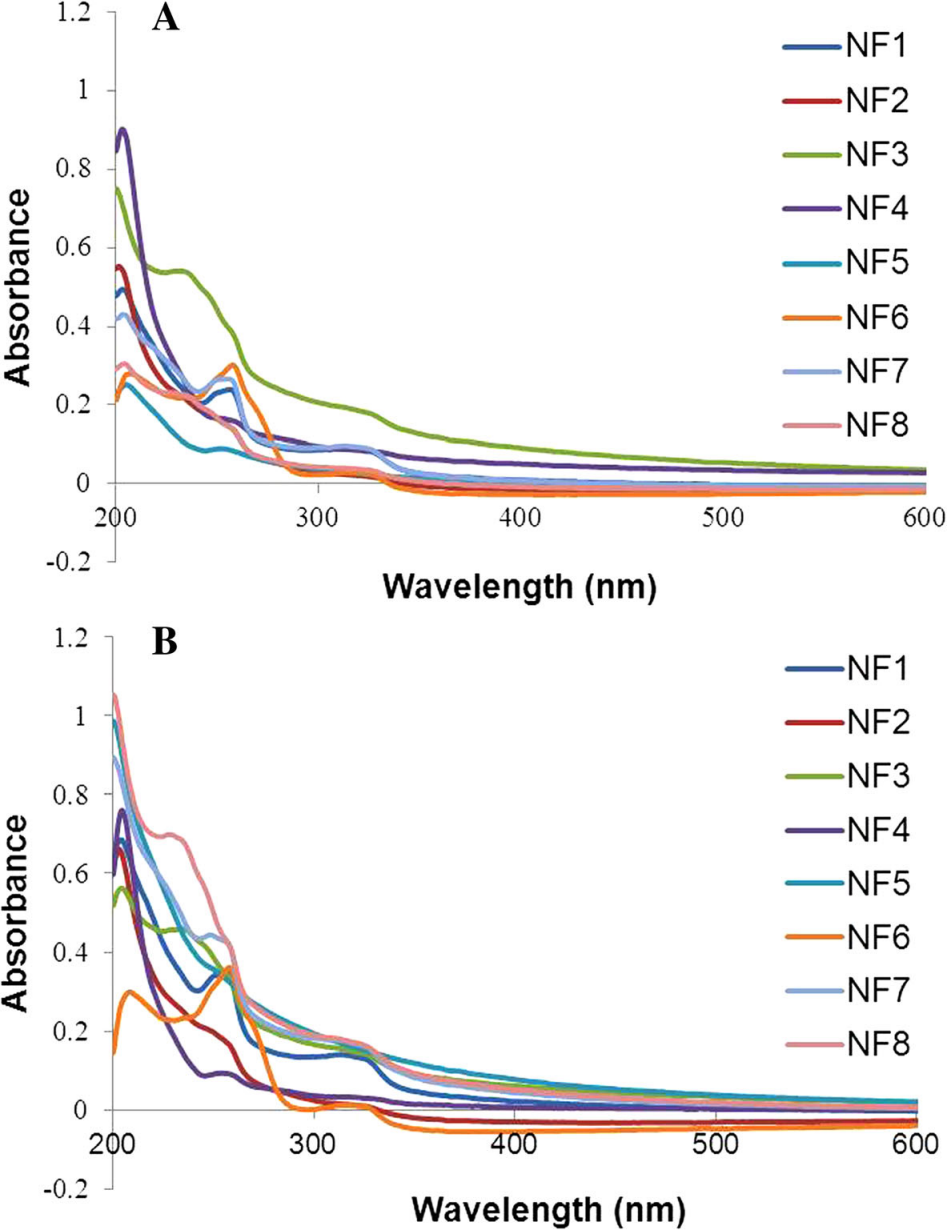
Absorption spectra of prepared nanoformulations. **a** At 0 day. **b** After 12 months

The absorption pattern of the nanoemulsions showed that all eight nalidixic acid diacyl and sulfonyl acyl derivatives entrapped in nanoemulsions were stable for more than 12 months when kept at different temperature conditions as depicted in Fig. 6a (representing zero day absorption spectra) and Fig. 6b (representing absorption spectra after more than 12 months). These results confirmed that ultrasonic waves used in the preparation of nanoemulsions and different storage environments had no deteriorating effect on the structure of active nanotized nalidixic acid derivatives synthesized in this study. The higher stability of active ingredient entrapped inside nanoformulations is concordant with the stability data reported by Behbahani et al. which highlighted that the ultrasonic energy had no destructive effects on the structure of nalidixic acid molecule [38].

Entrapment efficiency of nanoemulsions was determined by measuring the absorption of all eight derivatives at different concentrations using UV–Vis absorption spectrophotometer at 257, 205, 246, 205, 254, 256, 257, and 208 nm, respectively. The standard curve for each active ingredient was established by plotting absorption against concentration and entrapment efficiency of all nanoformulations was calculated from standard curve. The results showed in Table 1 indicated that all nanoformulations have more than 80% entrapment efficiency. Highest entrapment efficiency of 88% and 91% was observed with NF7and NF2, respectively.

**Fig. 7:**
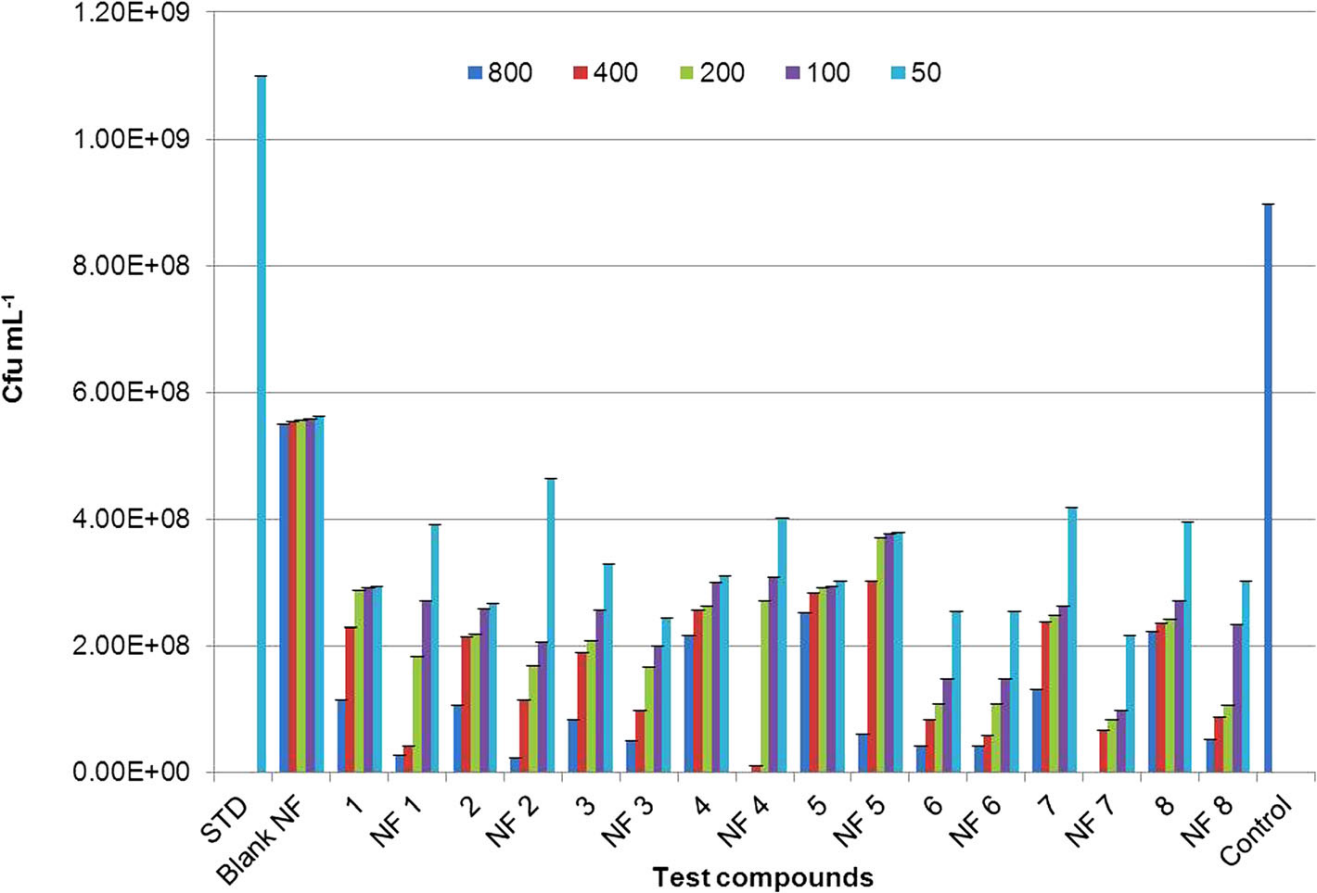
Effect of nanoformulation exposure on growth of *Staphylococcus aureus* (CFU mL^−1^)

**Fig. 8:**
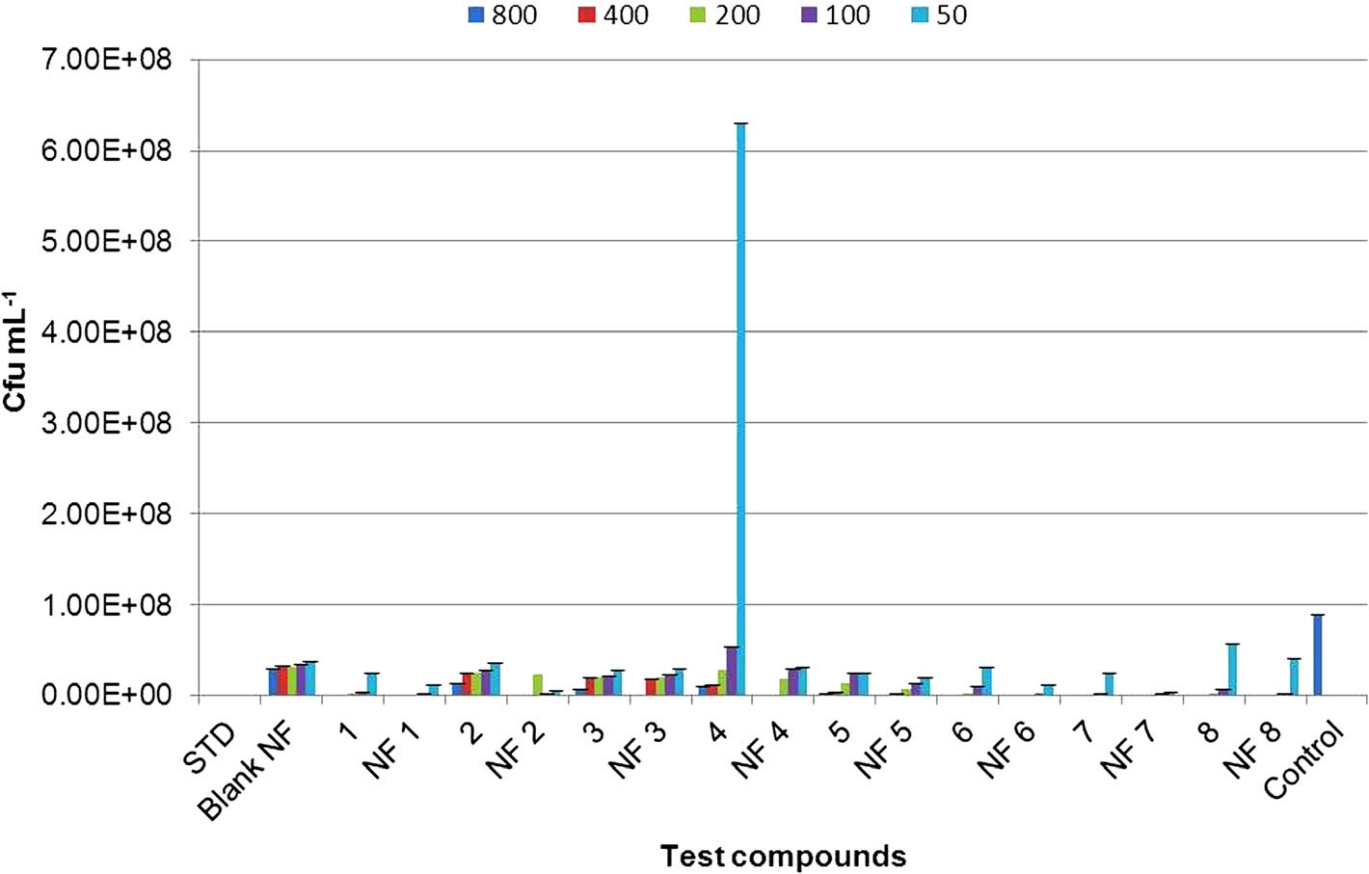
Effect of nanoformulation exposure on growth of *Bacillus subtilis* (CFU mL^−1^)

**Fig. 9:**
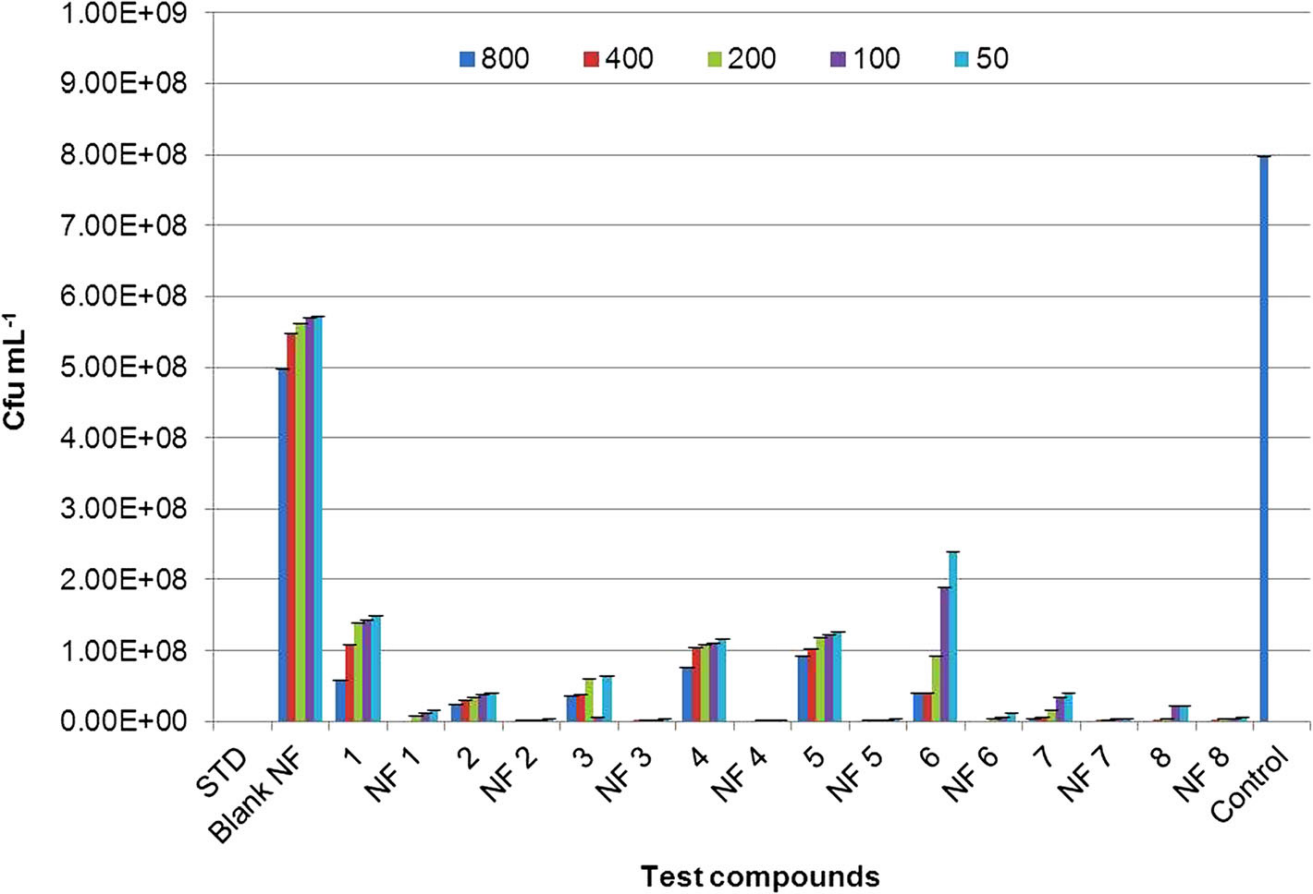
Effect of nanoformulation exposure on growth of *Pseudomonas aeruginosa* (CFU mL^−1^)

**Fig. 10:**
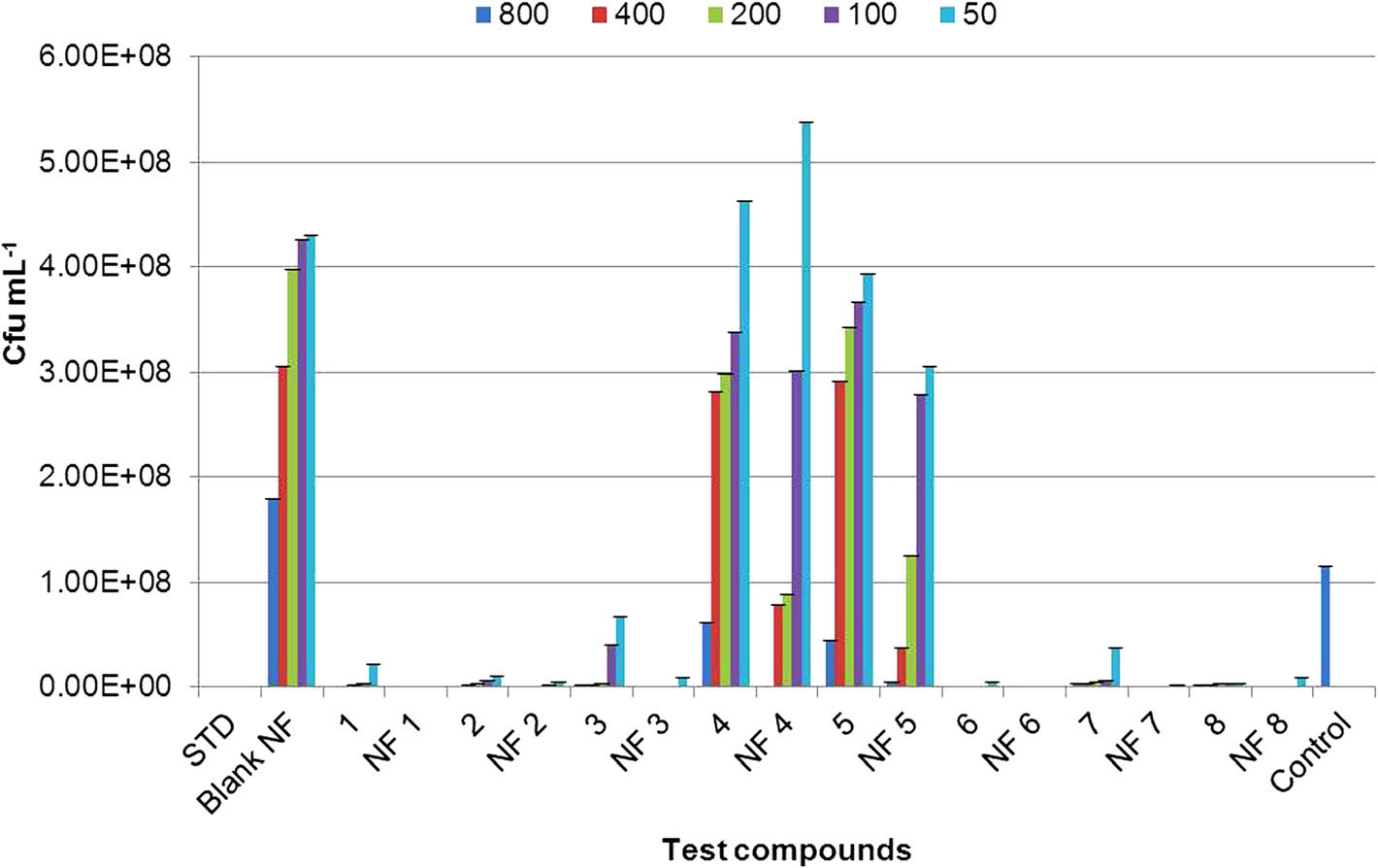
Effect of nanoformulation exposure on growth of *Acinetobacter baumannii* (CFU mL^−1^)

All nanoformulations and their corresponding non-nano derivatives were screened for their antibacterial activity using microbroth dilution method, against two Gram-positive bacteria, *S*. *aureus* and *B*. *subtilis*, and two Gram-negative bacteria, *P*. *aeruginosa* and *A*. *baumannii* at 800, 400, 200, 100, and 50 μg mL^−1^ concentrations. All nanoformulations displayed dose-dependent toxicity against test pathogens. Net change in OD_570_ after 18 h of exposure to nanoformulation on *S*. *aureus*, *B*. *subtilis*, *P*. *aeruginosa*, and *A*. *baumannii* were given in Additional file 1: Tables S2, S3, S4 and S5 respectively. Also, total number of viable cells present was confirmed by CFU analysis to determine the quantitative effect of the exposure on the bacterial cells (see Additional file 1: Figures S4 and S5, one representative image for Gram-positive and Gram-negative bacteria is shown). The results were given in Figs. 7, 8, 9, and 10 for *S*. *aureus*, *B*. *subtilis*, *P*. *aeruginosa*, and *A*. *baumannii*, respectively. The lowest concentration at which no perceptible colony was observed after 24-h incubation is considered as MIC of the test formulation. Careful analysis of results obtained showed that these nanoformulations displayed good toxicity at higher concentrations in the case of Gram-positive bacteria, *S*. a*ureus*, as compared to their corresponding non-nano derivatives. CFU results confirmed 100% growth inhibition of *S*. *aureus* at 800 μg mL^−1^ for nanoformulations 4 and 7. However, at lower concentrations, no significant toxicity was seen in comparison with non-nano derivatives. All nanoformulations displayed moderate to higher toxicity against *B*. *subtilis*. Nanoformulations 1, 4, 6, 7, and 8 displayed enhanced sensitivity even at 200 μg mL^−1^ concentrations as compared with their non-nano derivatives. Similarly, in the case of Gram-negative bacteria *P*. *aeruginosa* and *A*. *baumannii*, all nanoformulations displayed significant toxicity even at lower concentrations as compared to their non-nano derivatives. Nanoformulations 1, 4, and 6 displayed MIC at 200 μg mL^−1^against *P*. *aeruginosa* as evident from CFU results (Fig. 9) in comparison to their non-nano counterpart. Also, in the case of nanoformulations 2, 3, 5, and 7, no visible colonies were seen at 800 μg mL^−1^concentration. Maximum sensitivity was observed with NF1 and NF7 against *A*. *baumannii* which displayed complete inhibition at 100 μg mL^−1^ (Fig. 10). NF6 and 8 displayed MIC at 200 μg mL^−1^ concentration.

**Fig. 11:**
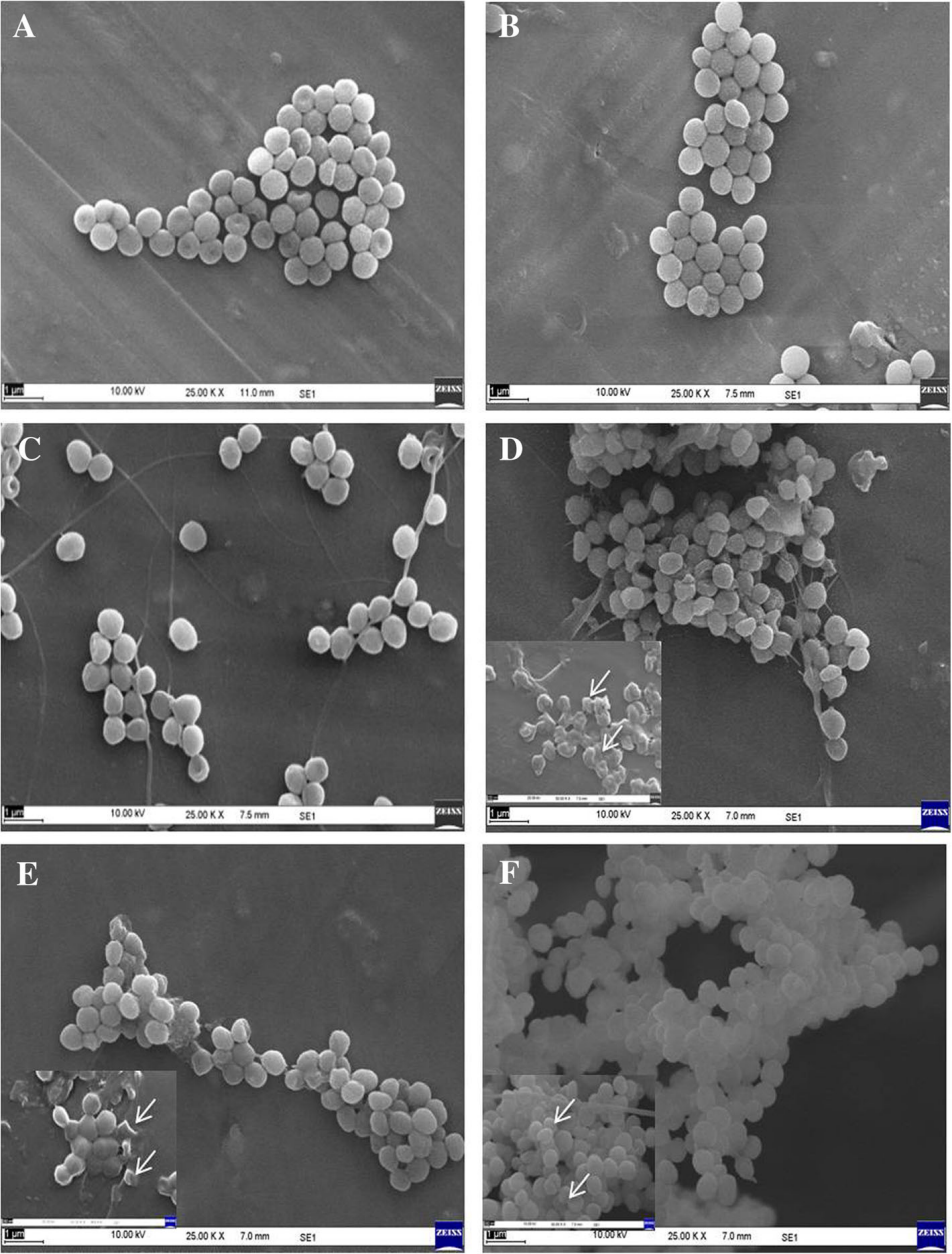
Study of antibacterial activity against *Staphylococcus aureus* by scanning electron microscopy (SEM). **a** Control. **b** Cells treated with 200 μg mL^−1^ nalidixic acid. **c** Cells treated with 200 μg mL^−1^ antibiotic Tetracycline. **d** Cells treated with 800 μg mL^−1^ of NF7. **e** Cells treated with 400 μg mL^−1^ of NF7. **f** Cells treated with 200 μg mL^−1^ of NF7 (high magnification images are shown at the bottom left corner taken at 200 nm scale bar and 50.00 kV). Unless otherwise indicated, the images are at 1 μm scale bar and 25.00 kV

**Fig. 12:**
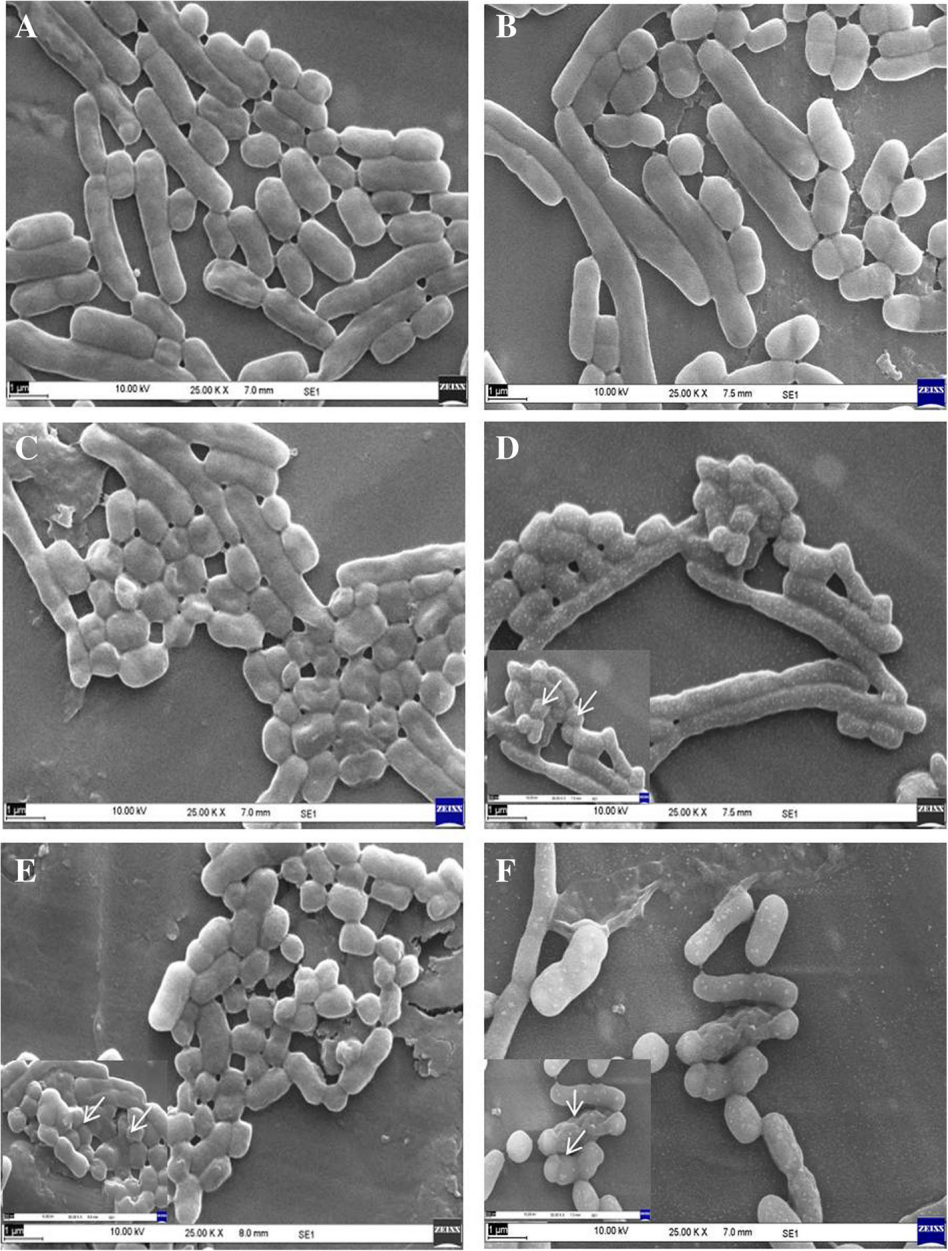
Study of antibacterial activity against *Acinetobacter baumannii* by scanning electron microscopy (SEM). **a** Control. **b** Cells treated with 200 μg mL^−1^ nalidixic acid. **c** Cells treated with 200 μg mL^−1^ Tetracycline. **d** Cells treated with 800 μg mL^−1^ of NF4. **e** Cells treated with 400 μg mL^−1^ of NF4. **f** Cells treated with 200 μg mL^−1^ of NF4 (high magnification images were shown at the corner taken at 200 nm scale bar and 50.00 kV). Unless otherwise indicated, the images are at 1 μm scale bar and 25.00 kV

SEM imaging was carried out to study the mode of antibacterial activity against Gram-positive bacteria *S*. *aureus* for NF7 and Gram-negative bacteria *A*. *baumannii*, for NF4 (Figs. 11 and 12 respectively) along with standard antibiotics tetracycline and parent molecule nalidixic acid. Results showed clear disruption of cell wall and formation of bleb-like structures on the cell wall of *S*. *aureus* (Fig. 11d–f). Such deformation was seen at all test concentrations, although prominent effect was observed at higher concentrations of 800 μg mL^−1^ and 400 μg mL^−1^. Such cellular deformities were not seen in the case of cells treated with nalidixic acid (Fig. 12d, f). Similarly, in the case of *A*. *baumannii*, disruption was easily seen even at lower concentrations of 200 μg mL^−1^ as compared to parent nalidixic acid showing negligible toxicity at same concentration (Fig. 12e). The bacterial cells exposed to nanoformulations showed obvious morphological changes as compared to untreated cells. Most of the treated bacterial cells became pitted, broken, or deformed. These observations further supported the results of microbroth dilution assay. Among all tested nanoformulations, sulfonyl acyl hydrazine-based nanoformulations, NF7 and NF8 displayed maximum sensitivity to the bacteria. These findings were further supported by the report that the compounds having sulfonyl acyl hydrazine group attached to their nuclei exhibited higher sensitivity to bacteria [39]. Furthermore, the mode of antibacterial activity and the mechanism by which these nanoemulsions exert their efficacy is attributed to their ability to adhere with the outer membranes of the microorganisms, due to the electrostatic interaction between the cationic charge of the nanoemulsion and the anionic charge on the microorganisms, eventually disrupting the membrane’s lipid bilayers and its cellular penetrability, leading to disruption of membrane thereby resulting in the wide-spectrum activity of such nanoemulsions as reported in the literature [40]. The results clearly indicated that nanotization of these active nalidixic acid-based molecules exhibited enhanced antibacterial activity when compared with respective non-nano counterparts.

All nanoformulations and their corresponding non-nano derivatives displayed virtuous sensitivity to *C*. *albicans*. However, the toxicity was more profound in the case of nanoformulations as depicted in Table 2. The maximum inhibitory activity was seen in case of nanoformulations 6, 7, and 8 showing 16, 15, and 14 mm inhibition zone at lowest concentration of 50 μg mL^−1^ respectively. On the other hand, *C*. *albicans* displayed no sensitivity to non-nano bromodiacyl hydrazine derivatives 4 and 5; consequently, their nanoformulations displayed relatively lower toxicity. The zone of inhibition for each treatment corresponding to nanoformulations and their non-nano derivatives is shown in Additional file 1: Figure S6. It was observed that both positive controls viz., Cycloheximide and Nystatin used in the assay showed absolutely no sensitivity to *C*. *albicans* at all test concentrations. Our results of *C*. *albicans* growth inhibition is in agreement with the previously reported literature where in ultrafine lipid nanocarriers can easily fuse with *Candida* cell membrane disturbing the membrane integrity and eventually resulting in loss of cell viability [41].

**Table 2:** Zone of inhibition observed for nalidixic acid derivatives and their relevant nanoformulation against *Candida albicans* (in mm)

Concentration (μgmL^-1^)	800	400	200	100	50
Compound					
1	20 ± 2.83	19 ± 1.41	15 ± 2.83	12 ± 1.41	–
NF1	23 ± 1.41	21 ± 2.83	20 ± 1.41	15 ± 1.41	10 ± 2.83
2	20 ± 1.41	20 ± 2.83	15 ± 1.41	10 ± 4.24	05 ± 1.41
NF2	24 ± 2.83	21 ± 2.83	15 ± 2.83	10 ± 0	07 ± 1.41
3	18 ± 2.83	1.5 ± 2.83	09 ± 1.41	–	–
NF3	23 ± 2.83	21 ± 1.41	15 ± 1.41	10 ± 0	–
4	–	–	–	–	–
NF4	06 ± 1.41	03 ± 1.41	–	–	–
5	–	–	–	–	–
NF5	03 ± 1.41	–	–	–	–
6	21 ± 1.41	17 ± 2.83	14 ± 1.41	12 ± 1.41	11 ± 1.41
NF6	24 ± 1.41	22 ± 1.41	19 ± 1.41	17 ± 1.41	16 ± 2.83
7	17 ± 1.41	16 ± 1.41	13 ± 2.83	10 ± 1.41	07 ± 1.41
NF7	20 ± 2.83	18 ± 1.41	18 ± 2.83	15 ± 2.83	15 ± 1.41
8	18 ± 1.41	16 ± 1.41	12 ± 1.41	10 ± 1.41	06 ± 1.41
NF8	20 ± 1.41	20 ± 1.41	17 ± 1.41	16 ± 1.41	14 ± 1.41
Cycloheximide	–	–	–	–	–
Nystatin	–	–	–	–	–
Control	–				

*NF* nanoformulation, – implies no inhibition

**Fig. 13:**
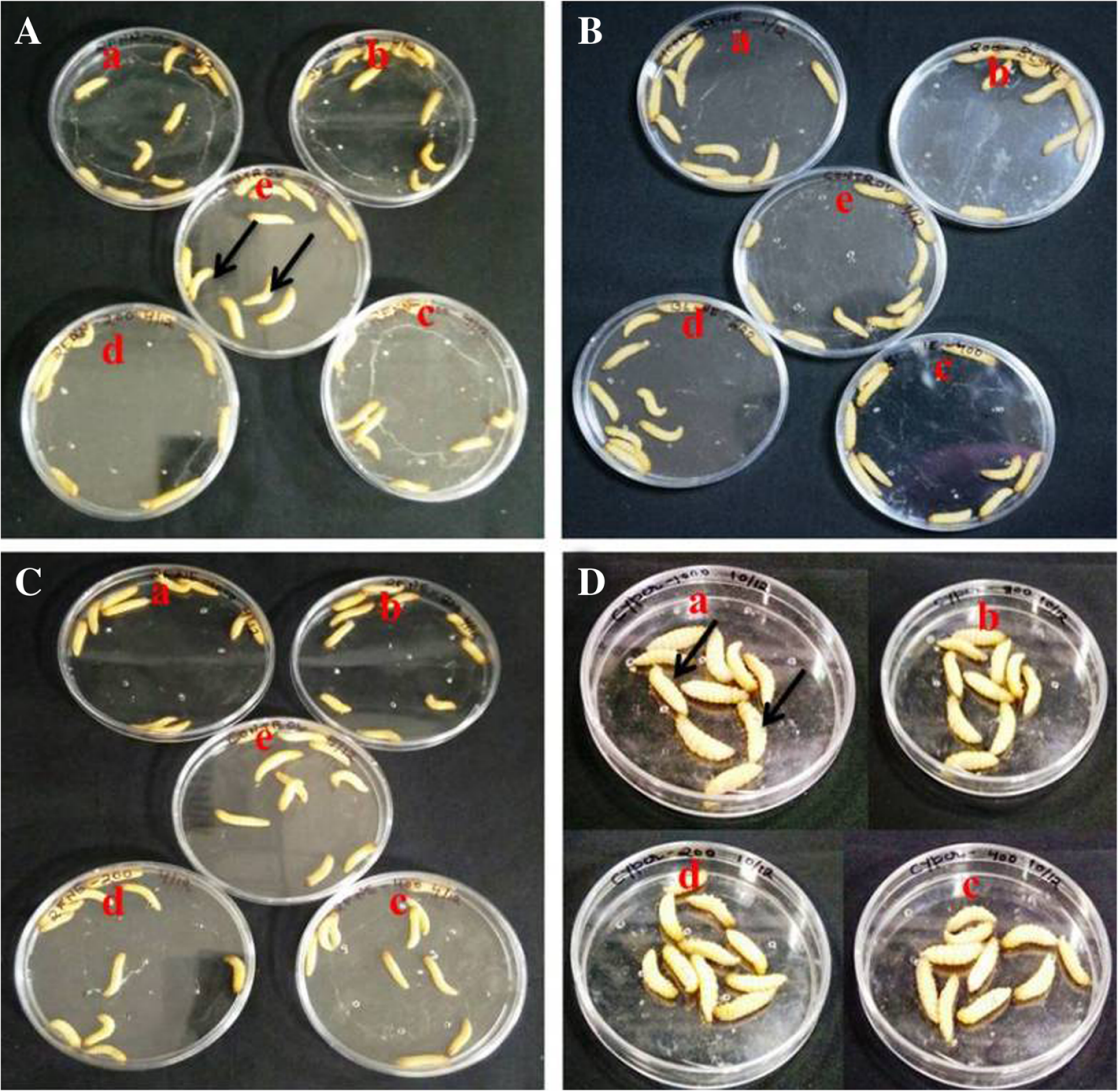
Biosafety study of nanoformulation exposure to *Galleria mellonella* larvae. This mammalian model was exposed to different concentrations (arrows are representing dead or living larvae). (a) 1000, (b) 800, (c) 400, (d) 200 ppm, (e) control. **a** Non-nano derivative. **b** Nanoformulation (only one representative image is shown). **c** Blank nanoformulation. **d** Cypermethrin 25EC

Toxicity studies reveled that all nanoformulations displayed no toxicity to *G*. *mellonella* larvae even after 72 h of treatment. Each treated larva displayed healthy growth and movement at highest concentration of 1 mg mL^−1^ (Fig. 13). Similar trend was observed with blank nanoformulation. However, with Cypermethrin 25EC, 100% mortality was observed immediately within 1 min of treatment. In Fig. 13d, dead larvae can easily be identified lying upside down. On the other hand in all other treatments, healthy moving larvae can be seen. To the best of our knowledge, biosafety study of nanoformulations based on nalidixic acid diacyl hydrazine and sulfonyl acyl hydrazine derivatives on *G*. *mellonella* larvae is reported for the first time in this paper.

## Conclusions

Globally, resistance to antibiotics is a major problem and innovative work is needed in the area to combat antimicrobial drug resistance. The results of the work presented here signify the potentials of nanotechnological applications for the revival of the quinolone antibiotic, nalidixic acid. All nanoformulations of nalidixic acid-based derivatives displayed high stability of more than 12 months at different storage conditions. Antibacterial assessment studies showed promising results compared with their non-nano derivatives on potential pathogenic bacteria, *S*. *aureus*, *B*. *subtilis*, *P*. *aeruginosa*, and *A*. *baumannii*. Also, biosafety studies on the mammalian model, *G*. *mellonella*, displayed no toxicity of all nanoformulations. To the best of our knowledge, we present here, the first report on the revival of nalidixic acid through the development of stable nanoformulations.

## Additional File

Additional file 1: **Figure S1.** Synthetic scheme. **Figure S2.** Particle size distribution of nanoformulations: a) NF 1, b) NF 2, c) NF 3, d) NF 4, d) NF 5, e) NF 6, f) NF 7 and g) NF 8. **Figure S3.** TEM images of nanoformulation a) NF1, b) NF2, c) NF3, d) NF4 and e) NF5 after 3 months storage at room temperature (representative images shown, scale bar is shown at the bottom left corner of each figure and varied between 20 nm to 100 nm). **Figure S4.** Antibacterial activity of nanoformulations and non-nano derivatives against *bacillus subtilis* using CFU per mL. a) 800, b) 400, c) 200, d) 100, e) 50μgmL^1^ and f) Control. **Figure S5.** Antibacterial activity of nanoformulations and non-nano derivatives against *Acinetobacter baumannii* using CFU per mL. a) 800, b) 400, c) 200, d) 100, e) 50μgmL^− 1^ and f) Control. **Figure S6.** Anticandida activity of nanoformulations (NF) along with their non-nano derivatives (NN) showing zone of inhibition at each test concentration in μgmL^− 1^. Table S1a Stability of prepared nanoformulations stored at 4 °C. **Table S1b.** Stability of prepared nanoformulations stored at 25 °C. **Table S2.** Net change in OD (average of 3 readings) at 570 nm after 18 h of exposure to nanoformulations on *Staphylococcus aureus*. **Table S3.** Net change in OD (average of 3 readings) at 570 nm after 18 h of exposure to nanoformulations on *Bacillus subtilis*. **Table S4.** Net change in OD (average of 3 readings) at 570 nm after 18 h of exposure to nanoformulations on *Pseudomonas aeruginosa*. **Table S5.** Net change in OD (average of 3 readings) at 570 nm after 18 h of exposure to nanoformulations on *Acinetobacter baumannii*. (DOCX 14298 kb)

## Abbreviations

CDCl_3_: Deuterated chloroform
CFU: Colony forming unit
CLSI: Clinical and laboratory standards institute
DI: Dispersity index
GRAS: Generally regarded as safe
MTT: (3-(4,5-dimethyl-2-thiazolyl)-2,5-diphenyl-2H-tetrazolium bromide)
NF: Nanoformulations
NMR: Nuclear magnetic resonance
SEM: Scanning electron microscopy
TEM: Transmission electron microscopy
TLC: Thin layer chromatography
TMS: Tetramethylsilane
UV-Vis: Ultraviolet-visible

## References

[1] Golkar Z, Bagazra O, Pace DG (2014). Bacteriophage therapy: a potential solution for the antibiotic resistance crisis. J Infect Dev Ctries.

[2] Sengupta S, Chattopadhyay MK, Grossart HP (2013). The multifaceted roles of antibiotics and antibiotic resistance in nature. Front Microbiol.

[3] Fair RJ, Tor Y (2014). Antibiotics and bacterial resistance in the 21st century. Perspect Medicin Chem.

[4] Wright GD (2014). Something new: revisiting natural products in antibiotic drug discovery. Can J Microbiol.

[5] Centers for Disease Control and Prevention, antibiotic/ antimicrobial resistance. Antibiotic resistance threats in the United States. 2013. http://www.cdc.gov/drugresistance/threat-report-2013. Accessed 19 Sept 2018

[6] Tanwar J, Das S, Fatima Z, Hameed S (2014). Multidrug resistance: an emerging crisis. Interdiscip Perspect Infect Dis.

[7] Ventola CL (2015). The antibiotic resistance crisis: part 1: causes and threats. Pharm and Ther.

[8] Davies J, Davies D (2010). Origins and evolution of antibiotic resistance. Microbiol Mol Biol Rev.

[9] World Health Organization, Antibiotic resistance, Fact sheet. 2016. https://www.who.int/mediacentre/factsheets/fs194/en/. Accessed 19 Sept 2018

[10] Hooper DC, Jacoby GA (2015). Mechanisms of drug resistance: quinolone resistance. Ann NY Acad Sci.

[11] Aldred KJ, Kerns RJ, Osheroff N (2014). Mechanism of quinolone action and resistance. Biochemist.

[12] Emmerson A. M. (2003). The quinolones: decades of development and use. Journal of Antimicrobial Chemotherapy.

[13] Andriole Vincent T. (2005). The Quinolones: Past, Present, and Future. Clinical Infectious Diseases.

[14] Drlica K, Hiasa H, Kerns R, Malik M, Mustaev A, Zhao X (2009). Quinolones: action and resistance updated. Curr Top Med Chem.

[15] Amin M, Mehdinejad M, Pourdangchi Z (2009). Study of bacteria isolated from urinary tract infections and determination of their susceptibility to antibiotics. Jundishapur J Microbiol.

[16] Zhu X, Radovic-Moreno AF, Wu J, Langer R, Shi J (2014). Nanomedicine in the management of microbial infection—overview and perspectives. NanoToday.

[17] Aggarwal N, Kumar R, Dureja P, Khurana JM (2011). Synthesis, antimicrobial evaluation and QSAR analysis of novel nalidixic acid based 1,2,4-triazole derivatives. Eur J Med Chem.

[18] Aggarwal N, Kumar R, Srivastava C, Durejaa P, Khurana JM (2014). Synthesis, biological activities and SAR studies of novel 1-Ethyl-7-methyl-4-oxo-1,4-dihydro-[1,8]naphthyridine-3-carboxylic acid based diacyl and sulfonyl acyl hydrazines. Pest Manag Sci.

[19] Pelgrift RY, Friedman AJ (2013). Nanotechnology as a therapeutic tool to combat microbial resistance. Adv Drug Deliv Rev.

[20] Parveen S, Misra R, Sahoo SK (2012). Nanoparticles: a boon to drug delivery, therapeutics, diagnostics and imaging. Nanomedicine.

[21] Rudramurthy GR, Swamy MK, Sinniah UR, Ghasemzadeh A (2016). Nanoparticles: alternatives against drug resistant pathogenic microbes. Molecules.

[22] Shimanovich U, Gedanken A (2016). Nanotechnology solutions to restore antibiotic activity. J Mater Chem B.

[23] Bhatia S (2016). Natural polymer drug delivery systems, nanoparticles, plants and algae.

[24] Chen G, Roy I, Yang C, Prasad PN (2016). Nanochemistry and nanomedicine for nanoparticle-based diagnostics and therapy. Chem Rev.

[25] Safari J, Zarnegar Z (2014). Advanced drug delivery systems: nanotechnologies of health design a review. J Saudi Chem Soc.

[26] Food additives status list, United States Food and drug Administration. 2018. https://www.fda.gov/food/ingredientspackaginglabeling/foodadditivesingredients/ucm091048.htm. Accessed 19 Sept 2018.

[27] Gupta A, Eral HB, Hatton TA, Doyle PS (2016). Nanoemulsions: formation, properties and applications. Soft Matter.

[28] Gulati M, Grover M, Singh M, Singh S (1998). Study of azathioprine encapsulation into liposomes. J Microencapsul.

[29] Clinical and Laboratory Standards Institute (2015) Methods for dilution antimicrobial susceptibility tests for bacteria that grow aerobically, Approved standard, 10th edn. CLSI document, M07-A10, Wayner https://clsi.org/media/1632/m07a10_sample.pdf. Accessed 19 Sept 2018

[30] Duarte MC, Figueira GM, Sartoratto A, Rehder VLG, Delarmelina C (2005). Anticandida activity of Brazilian medicinal plants. J Ethnopharmacol.

[31] Desbois AP, Coote PJ, Laskin AI, Sariaslani S, Gadd GM (2012). Utility of greater wax moth larva (*Galleria mellonella*) for evaluating the toxicity and efficacy of new antimicrobial agents. Advances in applied microbiology.

[32] Ramarao N, Nielsen-Leroux C, Lereclus D (2012). The insect *Galleria mellonella* as a powerful infection model to investigate bacterial pathogenesis. J Vis Exp.

[33] Yang HF, Pan AJ, Hu LF, Liu YY, Cheng J, Ye Y, Li JB (2017). *Galleria mellonella* as an in vivo model for assessing the efficacy of antimicrobial agents against enterobacter cloacae infection. J MicrobioImmunol Infect.

[34] Tsai CJY, Loh JMS, Proft T (2016). *Galleria mellonella* infection models for the study of bacterial diseases and for antimicrobial drug testing. Virulence.

[35] Velikova N, Kavanagh K, Wells JM (2016). Evaluation of *Galleria mellonella* larvae for studying the virulence of Streptococcus suis. BMC Microbiolo.

[36] Kumar P, Ganguly S, Somvanshi VS (2015). Identification of virulent entomopathogenic nematode isolates from a countrywide survey in India. Int J Pest Manage.

[37] Behbahani GR, Sadr MH, Nabipour H, Oftadeh M, Rafiei S, Barzegar L (2013). Preparation of nanonalidixic acid and study of its biological properties. Open Access Scientific Reports.

[38] Bernardi DS, Pereira TA, Maciel NR, Bortoloto J, Viera GS, Oliveira GC, Rocha-Filho PA (2011). Formation and stability of oil-in-water nanoemulsions containing rice bran oil: in vitro and in vivo assessments. J Nanobiotech.

[39] Zareef M, Iqbal R, Mirza B, Khan KN, Manan A, Asim F, Khan SW (2008). Synthesis and antimicrobial activity of some derivatives of acylhydrazine including novel benzene diazasulfonamides. ARKIVOC.

[40] Hwang YY, Ramalingam K, Bienek DR, Lee V, You T, Alvarez R (2013). Antimicrobial activity of nanoemulsion in combination with cetylpyridinium chloride in multidrug-resistant *Acinetobacter baumannii*. Antimicrob Agents Chemother.

[41] Giongo JL, Vaucher RA, Fausto VP, Quatrin PM, Lopes LQS, Santos RCV, Gundel A, Gomes P, Steppe M (2016). Anti-Candida activity assessment of Pelargonium graveolens oil free and nanoemulsion in biofilm formation in hospital medical supplies. Microb Pathog.

