# Supplementary material for: Resurrection of Nalidixic Acid: Evaluation of Water-Based Nanoformulations as Potential Nanomedicine
Source: Nanoscale Res Lett. 2018 Sep 24;13:298. doi: 10.1186/s11671-018-2718-8 (PMC6153259; doi:10.1186/s11671-018-2718-8)
Supplement: Supplementary file 1 — Figure S1. Synthetic scheme. Figure S2. Particle size distribution of nanoformulations: a) NF 1, b) NF 2, c) NF 3, d) NF 4, d) NF 5, e) NF 6, f) NF 7 and g) NF 8. Figure S3. TEM images of nanoformulation a) NF1, b) NF2, c) NF3, d) NF4 and e) NF5 after 3 months storage at room temperature (representative images shown, scale bar is shown at the bottom left corner of each figure and varied between 20 nm to 100 nm). Figure S4. Antibacterial activity of nanoformulations and non-nano derivatives against bacillus subtilis using CFU per mL. a) 800, b) 400, c) 200, d) 100, e) 50μgmL1 and f) Control. Figure S5. Antibacterial activity of nanoformulations and non-nano derivatives against Acinetobacter baumannii using CFU per mL. a) 800, b) 400, c) 200, d) 100, e) 50μgmL− 1 and f) Control. Figure S6. Anticandida activity of nanoformulations (NF) along with their non-nano derivatives (NN) showing zone of inhibition at each test concentration in μgmL− 1. Table S1a Stability of prepared nanoformulations stored at 4 °C. Table S1b. Stability of prepared nanoformulations stored at 25 °C. Table S2. Net change in OD (average of 3 readings) at 570 nm after 18 h of exposure to nanoformulations on Staphylococcus aureus. Table S3. Net change in OD (average of 3 readings) at 570 nm after 18 h of exposure to nanoformulations on Bacillus subtilis. Table S4. Net change in OD (average of 3 readings) at 570 nm after 18 h of exposure to nanoformulations on Pseudomonas aeruginosa. Table S5. Net change in OD (average of 3 readings) at 570 nm after 18 h of exposure to nanoformulations on Acinetobacter baumannii. (DOCX 14298 kb) [file 11671_2018_2718_MOESM1_ESM.docx]

**Additional file 1**

**Resurrection of Nalidixic acid: Evaluation of Water-Based Nanoformulations as Potential Nanomedicine**

**Alka Pandey^1^, Nisha Aggarwal^2^, Alok Adholeya^1^ and Mandira Kochar^1^**

^1^TERI-Deakin Nanobiotechnology Centre, TERI Gram, The Energy and Resources Institute, Gual Pahari, Gurugram, Haryana, India.

^2^Department of Chemistry, Sri Aurobindo College, University of Delhi, New Delhi, India

***Corresponding author**. TERI-Deakin Nanobiotechnology Centre, TERI Gram, The Energy and Resources Institute, Gual Pahari, Gurugram 122003, Haryana, India. Fax: +91 011 24682144. *E-mail address:* [mandira.malhotra@gmail.com](mailto:mandira.malhotra@gmail.com), [mandira.kochar@teri.res.in](mailto:mandira.kochar@teri.res.in) (Mandira Kochar)


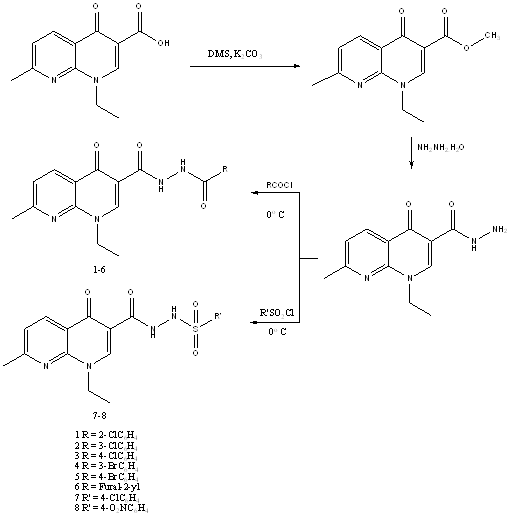


**Figure. S1.** Synthetic scheme

**Z-Average (d.nm): 138.41, DI: 0.288**

**g)**

**Z-Average (d.nm): 42.47, DI: 0.339**

**Z-Average (d.nm): 62.56, DI: 0.394**

**h)**

**a)**

**Z-Average (d.nm): 51.98, DI: 0.324**

**b)**

**Z-Average (d.nm): 70.42, DI: 0.337**

**c)**

**Z-Average (d.nm): 47.83, DI: 0.284**

**d)**

**e)**

**f)**

**Z-Average (d.nm): 76.56, DI: 0.194**

**Z-Average (d.nm): 54.67, DI: 0.226**

**Figure. S 2.** Particle size distribution of nanoformulations: a) NF 1, b) NF 2, c) NF 3, d) NF 4, d) NF 5, e) NF 6, f) NF 7 and g) NF 8.


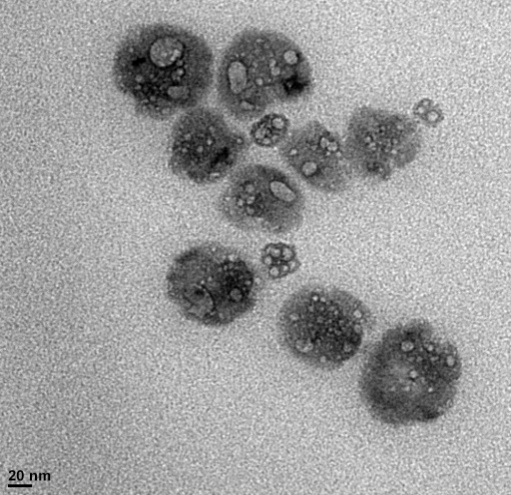

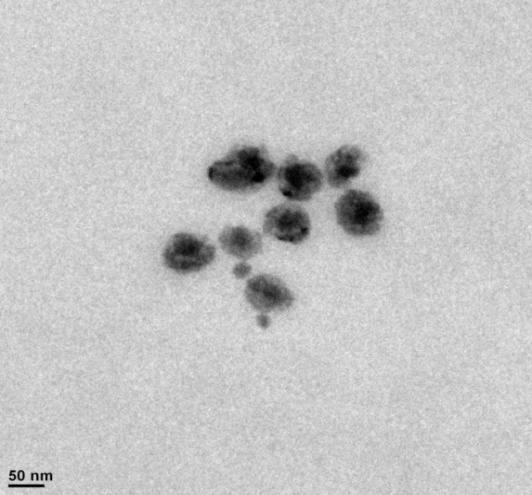

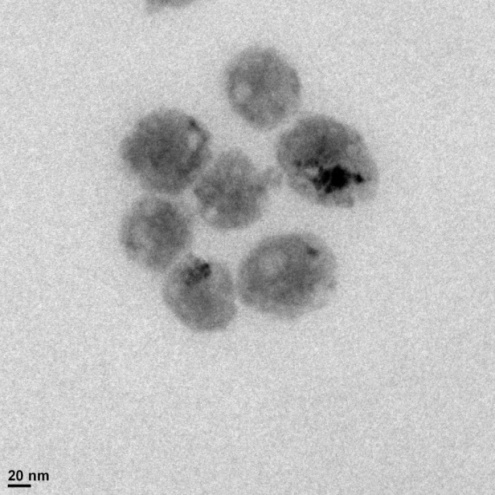

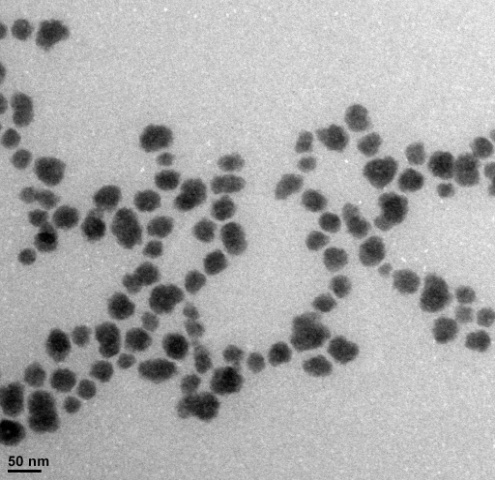

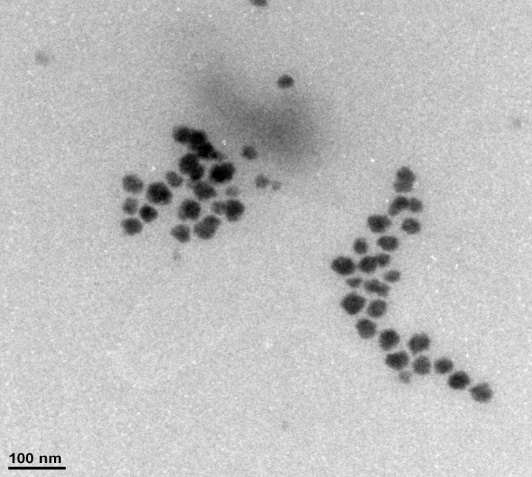


**d)**

**a)**

**b)**

**c)**

**e)**

**Figure. S3.** TEM images of nanoformulation a) NF1, b) NF2, c) NF3, d) NF4 and e) NF5 after three months storage at room temperature (representative images shown, scale bar is shown at the bottom left corner of each figure and varied between 20nm to 100nm).


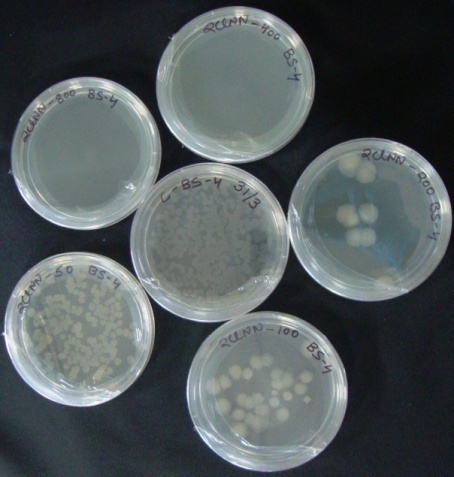

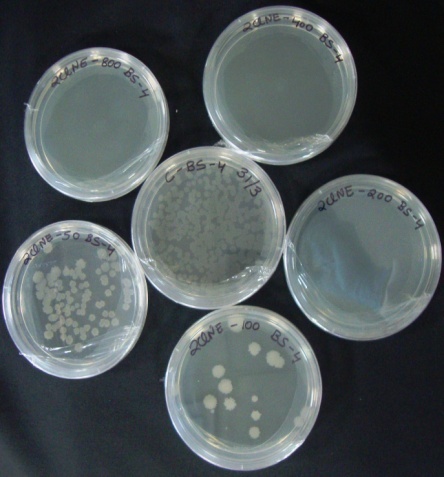

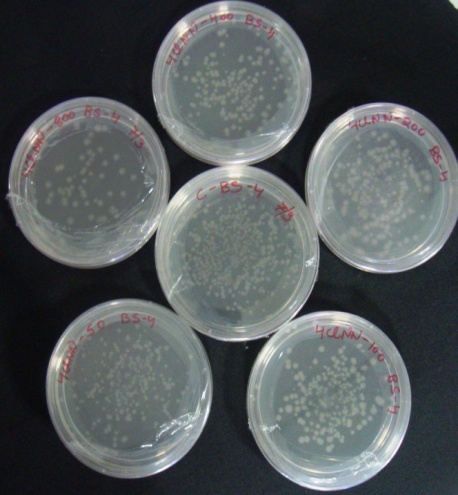

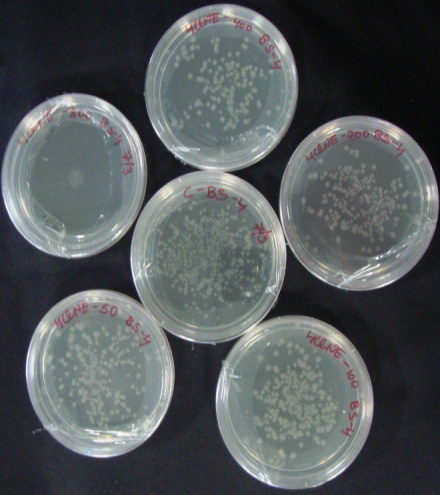

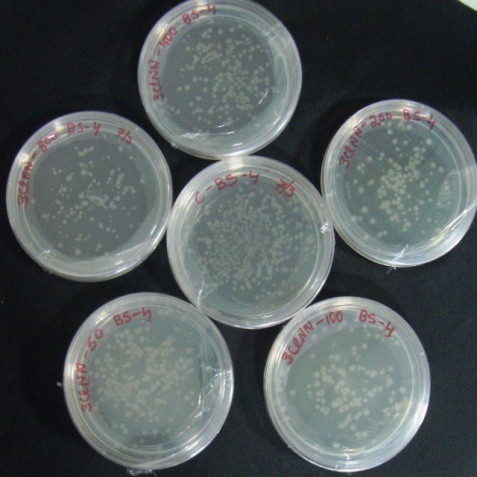

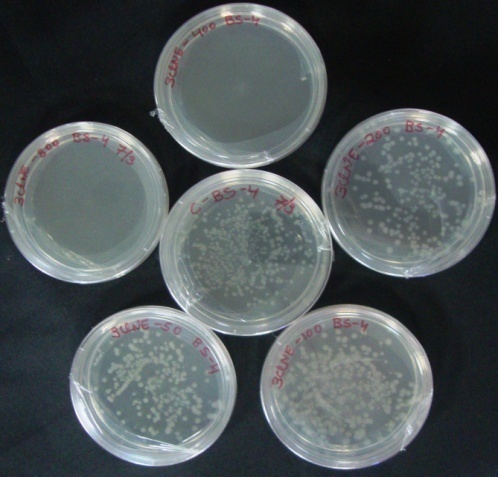

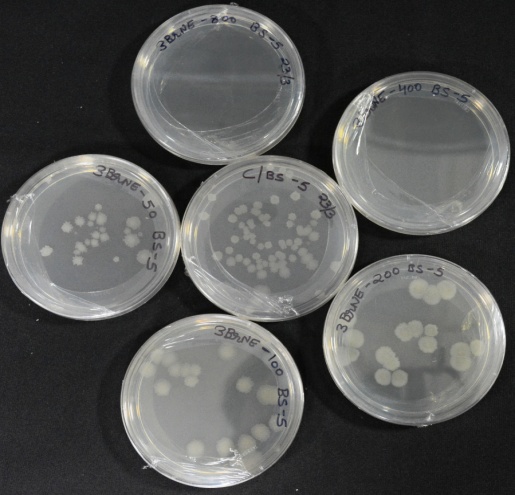

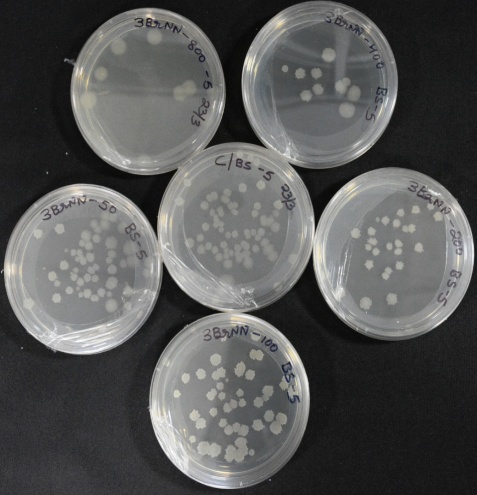


**a**

**b**

**c**

**d**

**e**

**f**

**a**

**b**

**c**

**d**

**e**

**f**

**a**

**b**

**c**

**d**

**e**

**f**

**a**

**b**

**c**

**d**

**e**

**f**

**a**

**b**

**c**

**d**

**e**

**f**

**a**

**b**

**c**

**d**

**e**

**f**

**a**

**b**

**c**

**d**

**e**

**f**

**a**

**b**

**c**

**d**

**e**

**f**

1

NF1

2

NF2

3

NF3

4

NF4


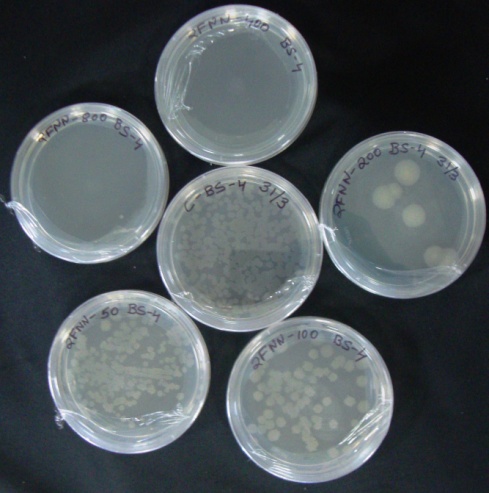

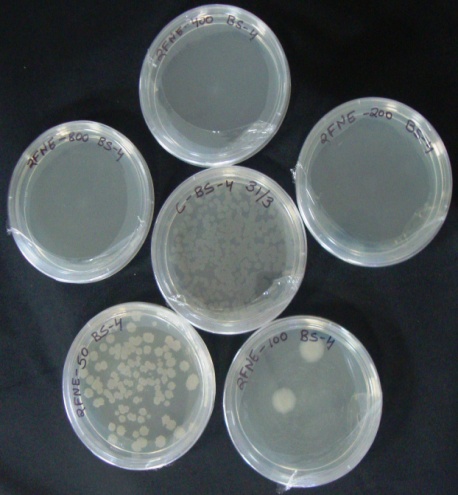

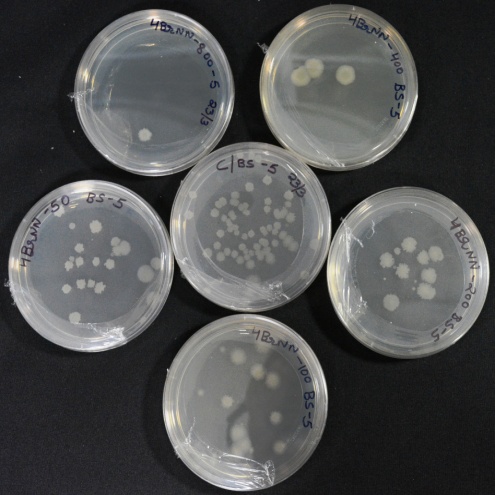

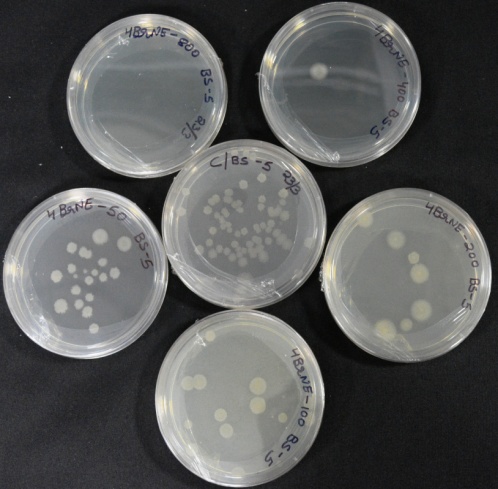

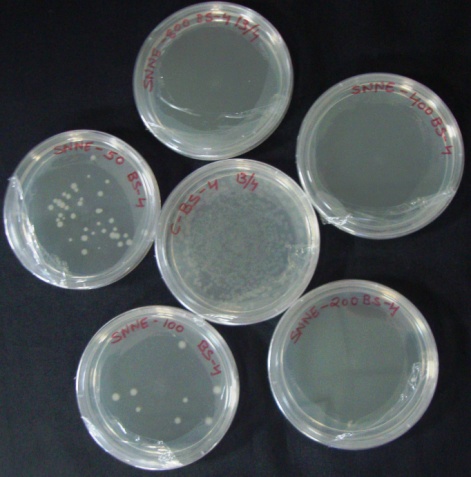

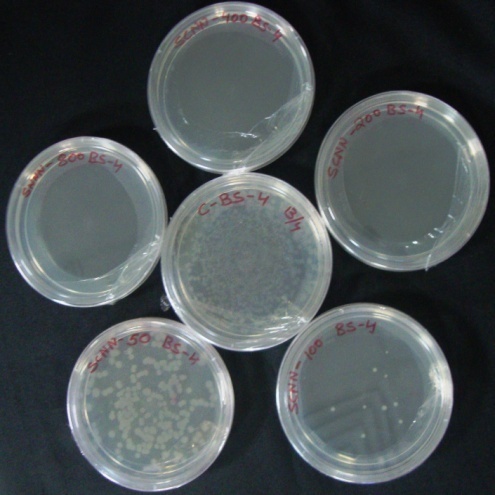

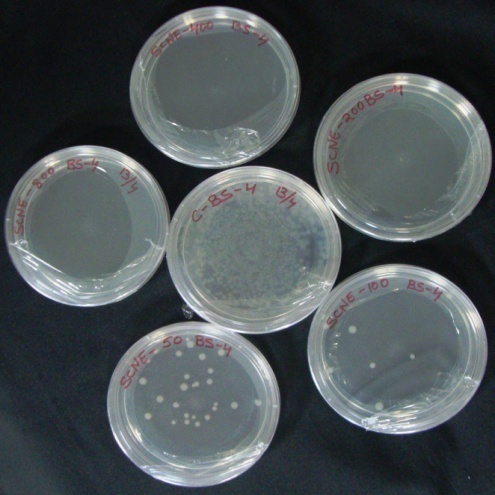

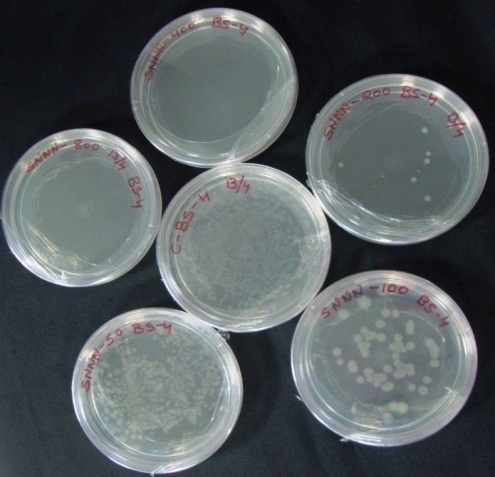


**a**

**b**

**c**

**d**

**e**

**f**

**a**

**b**

**c**

**d**

**e**

**f**

**a**

**b**

**c**

**d**

**e**

**f**

**a**

**b**

**c**

**d**

**e**

**f**

**a**

**b**

**c**

**d**

**e**

**f**

**a**

**b**

**c**

**d**

**e**

**f**

**a**

**b**

**c**

**d**

**e**

**f**

**a**

**b**

**c**

**d**

**e**

**f**

5

NF5

6

NF6

7

NF7

8

NF8


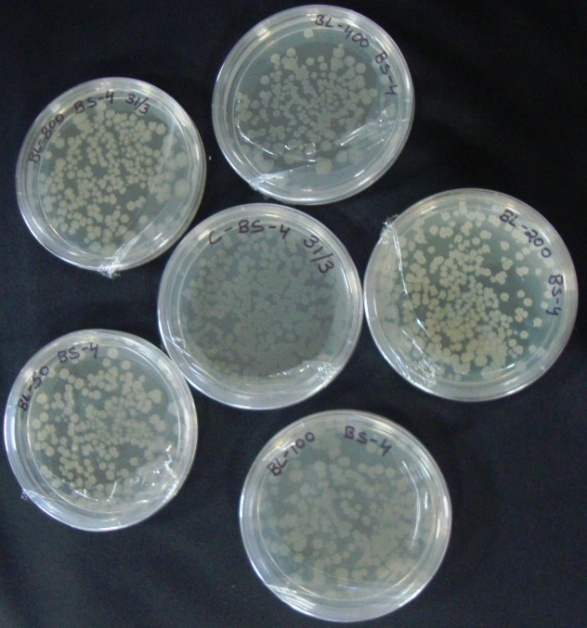

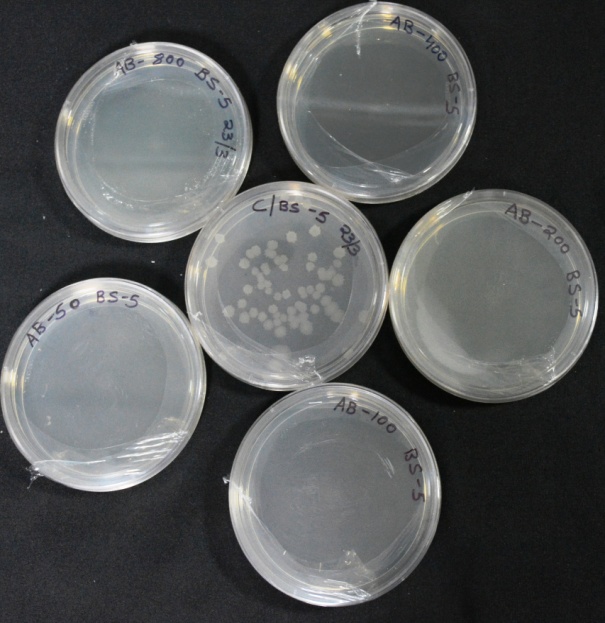


Blank Nanoformulation

Tetracycline

**a**

**b**

**c**

**d**

**e**

**f**

**a**

**b**

**c**

**d**

**e**

**f**

**Figure. S4.** Antibacterial activity of nanoformulations and non-nano derivatives against *bacillus subtilis* using CFU per mL. a) 800, b) 400, c) 200, d) 100, e) 50µgmL^1^ and f) Control.


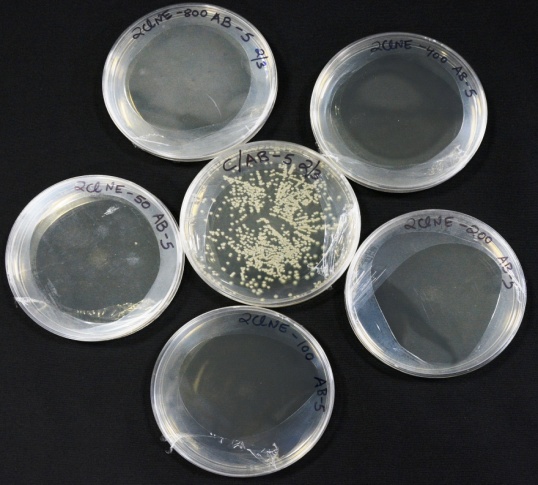

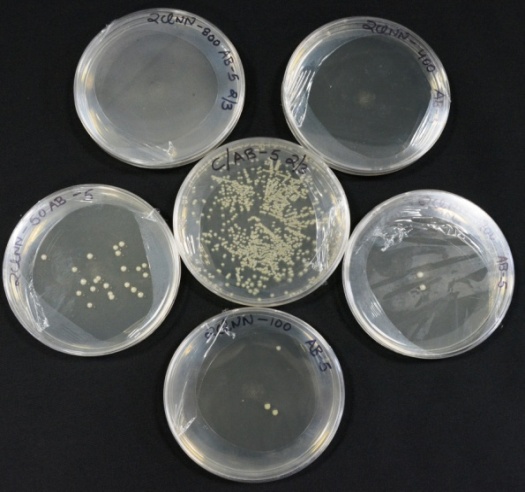


**a**

**b**

**c**

**d**

**e**

**a**

**b**

**c**

**d**

**e**

**f**

**f**


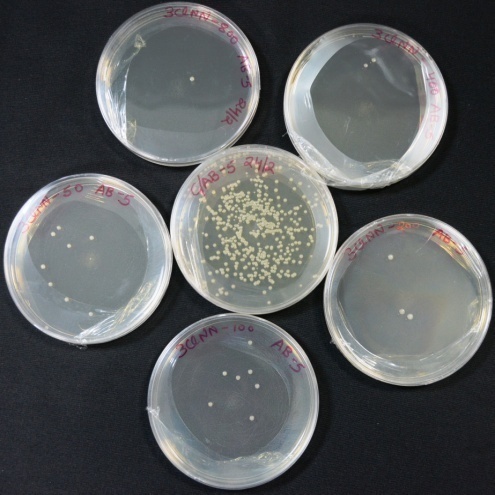

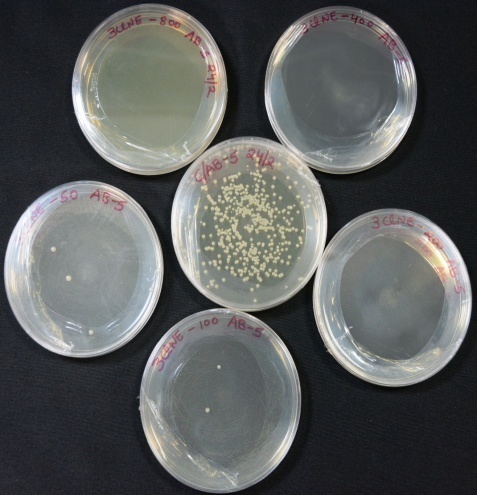

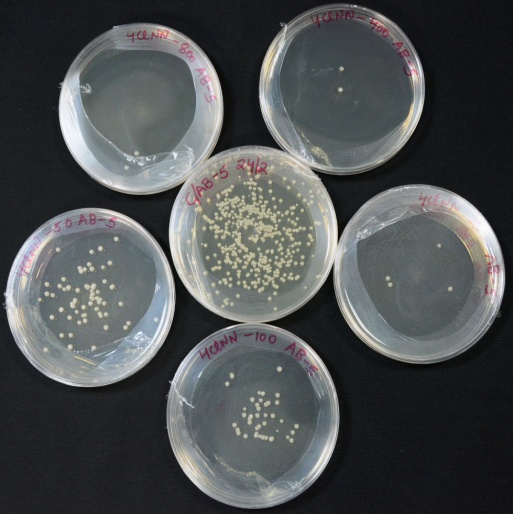

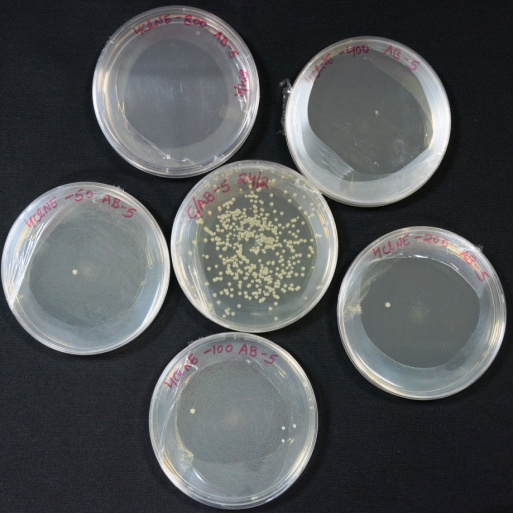

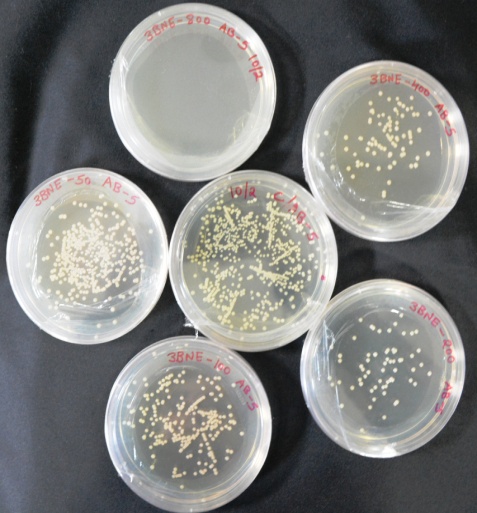

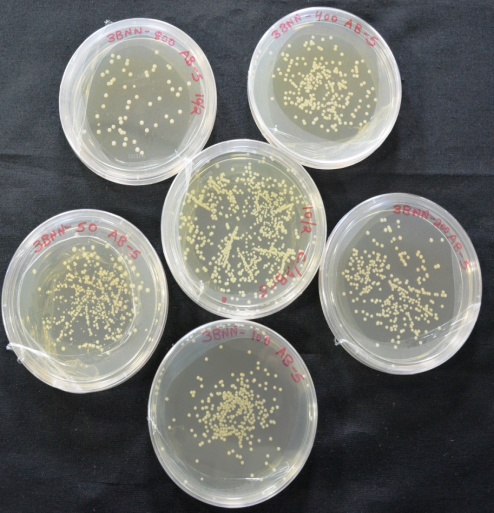


1

NF1

2

NF2

3

NF3

4

NF4

**a**

**b**

**c**

**d**

**e**

**f**

**a**

**b**

**c**

**d**

**e**

**f**

**a**

**b**

**c**

**d**

**e**

**f**

**a**

**b**

**c**

**d**

**e**

**f**

**a**

**b**

**c**

**d**

**e**

**f**

**a**

**b**

**c**

**d**

**e**

**f**


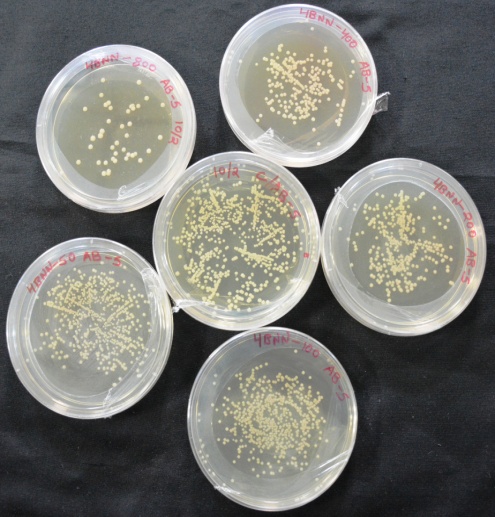

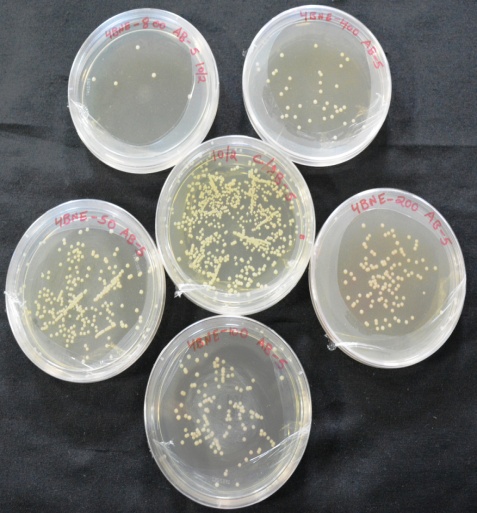

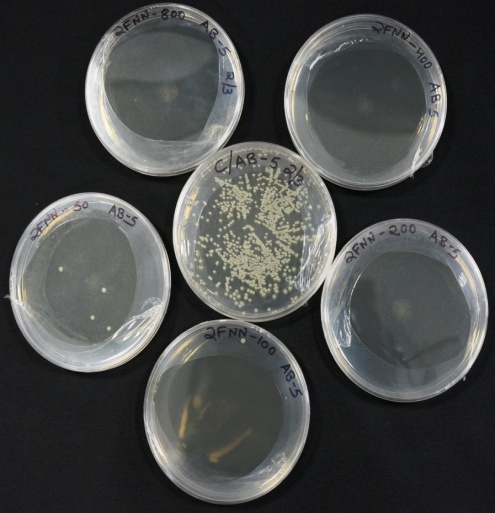

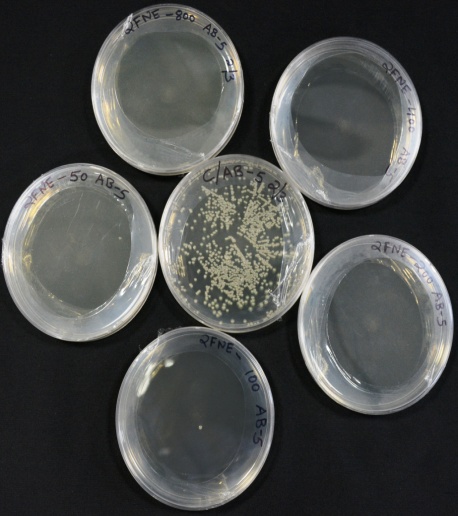

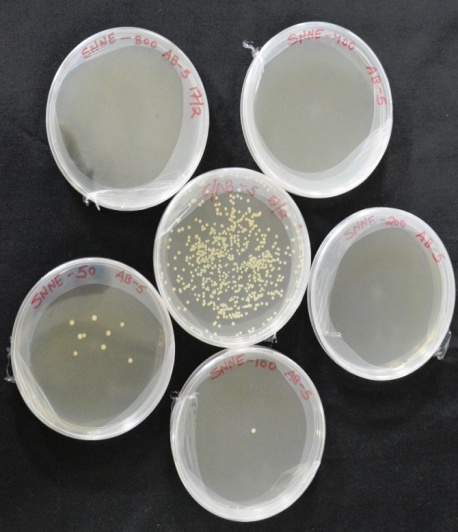

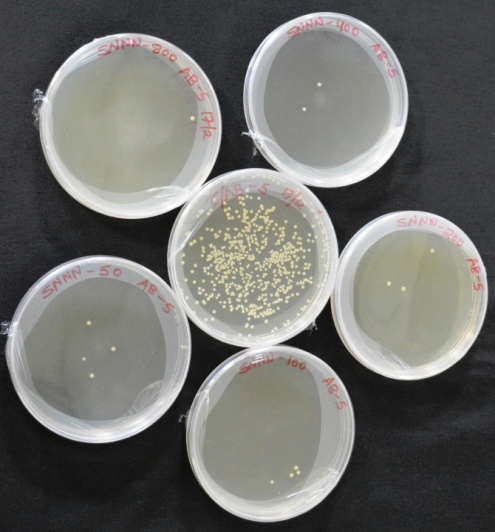

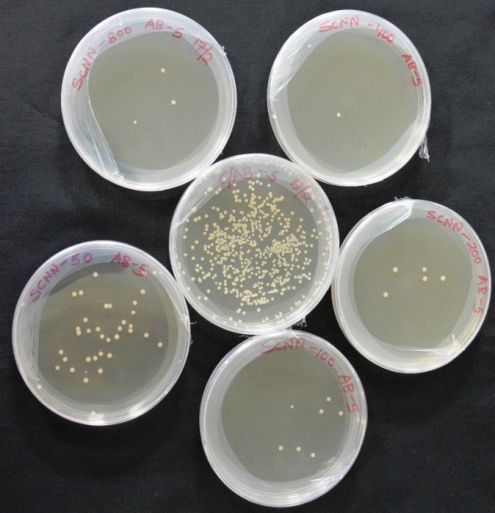

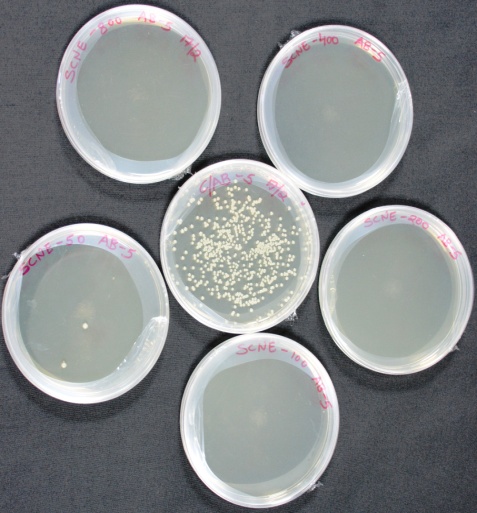


5

NF5

6

NF6

7

NF7

8

NF8

**a**

**b**

**c**

**d**

**e**

**f**

**a**

**b**

**c**

**d**

**e**

**f**

**a**

**b**

**c**

**d**

**e**

**f**

**a**

**b**

**c**

**d**

**e**

**f**

**a**

**b**

**c**

**d**

**e**

**f**

**a**

**b**

**c**

**d**

**e**

**f**

**a**

**b**

**c**

**d**

**e**

**f**

**a**

**b**

**c**

**d**

**e**

**f**


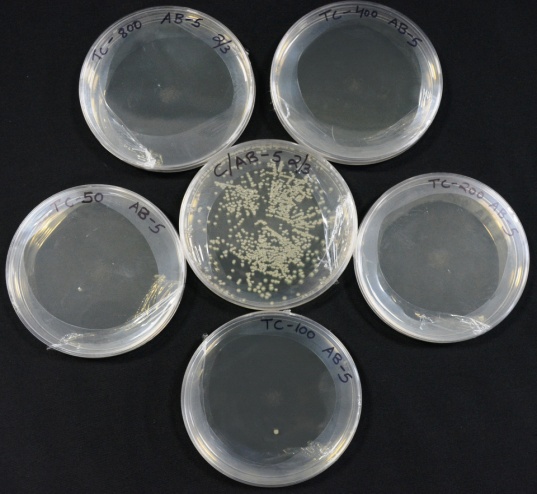

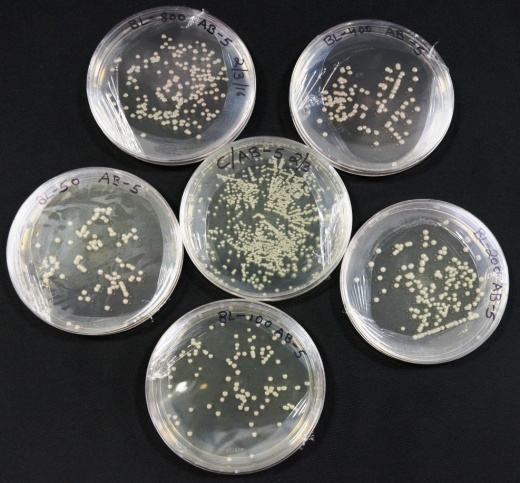


**a**

**b**

**c**

**d**

**e**

**f**

**a**

**b**

**c**

**d**

**e**

**f**

Blank Nanoformulation

Tetracycline

**Figure.S5.** Antibacterial activity of nanoformulations and non-nano derivatives against *Acinetobacter baumannii* using CFU per mL. a) 800, b) 400, c) 200, d) 100, e) 50µgmL^-1^ and f) Control.

**a) NF1 - 2ClNE**


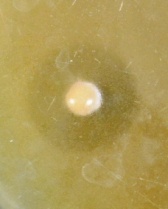

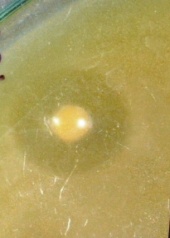

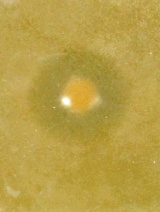

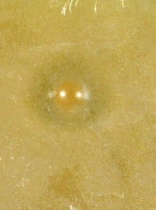

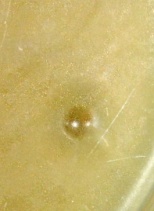


800 400 200 100 50

**2ClNN**


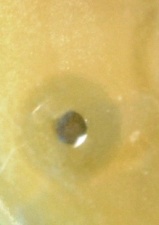

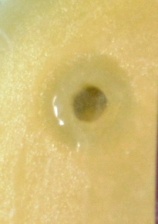

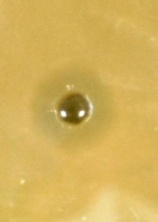

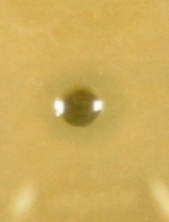

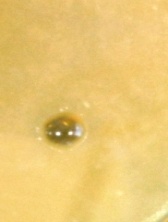


800 400 200 100 50

**b) NF 2 - 3ClNE**


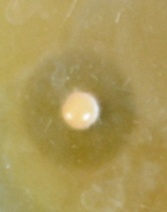

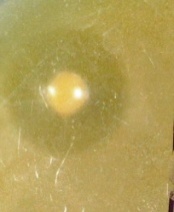

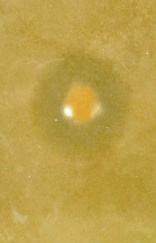

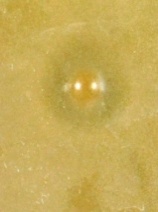

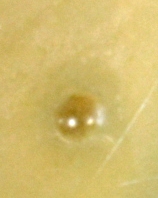


800 400 200 100 50

**3ClNN**


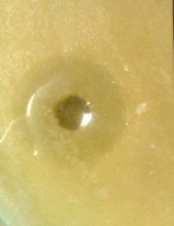

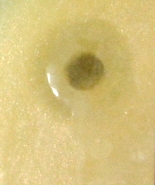

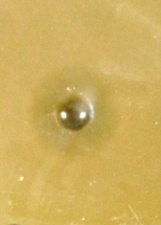

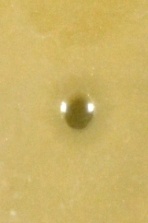

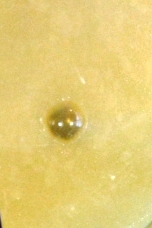


800 400 200 100 50

**c) NF3 - 4ClNE**


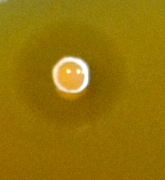

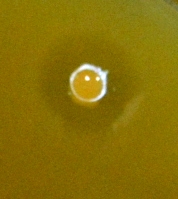

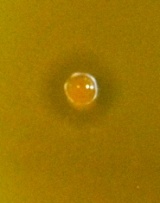

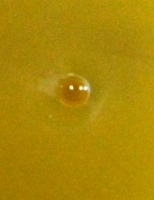

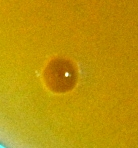


800 400 200 100 50

**4ClNN**


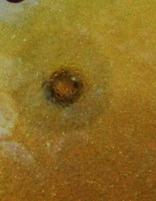

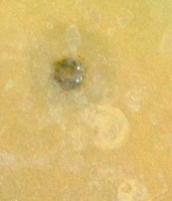

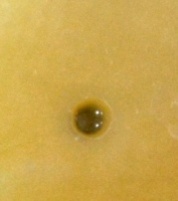

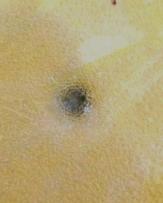

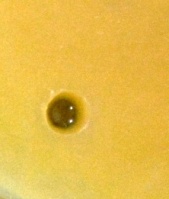


800 400 200 100 50

**d) NF 4 - 3BrNE**


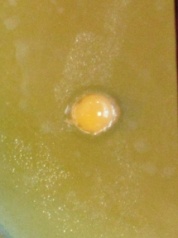

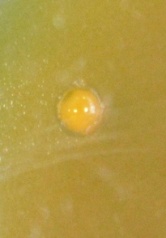

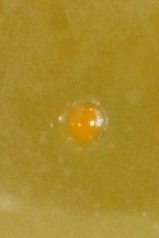

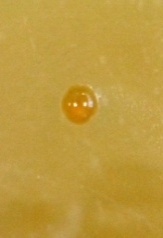

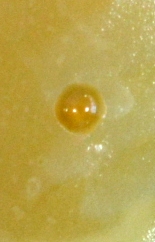


800 400 200 100 50

**3BrNN**


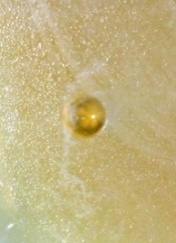

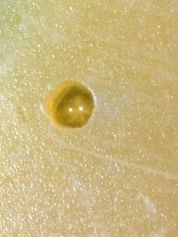

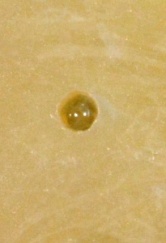

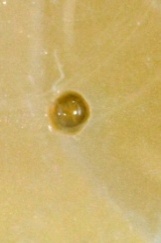

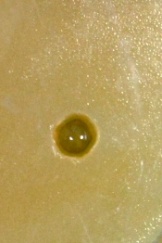


800 400 200 100 50

**e) NF 5 - 4BrNE**


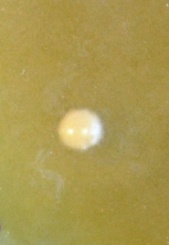

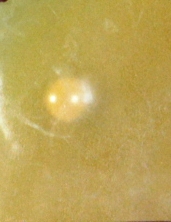

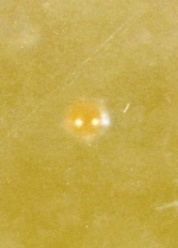

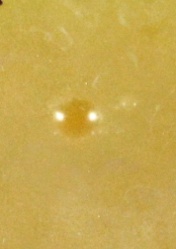

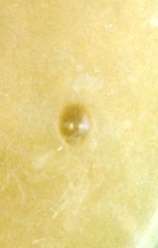


800 400 200 100 50

**4BrNN**


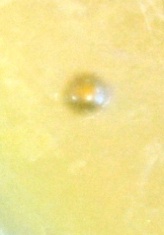

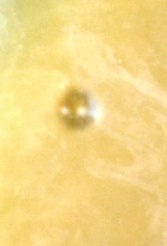

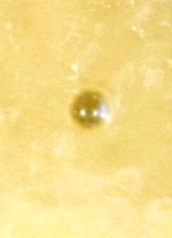

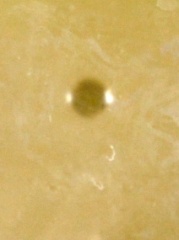

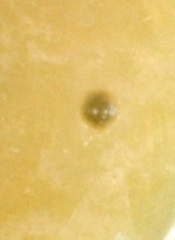


800 400 200 100 50

**f) NF6 - 2FNE**


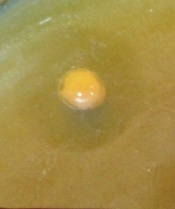

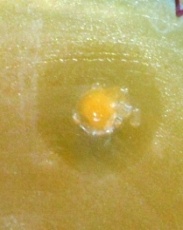

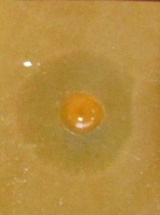

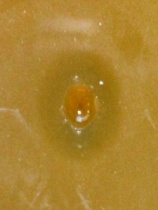

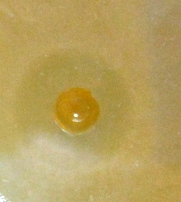


800 400 200 100 50

**2FNN**


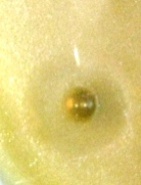

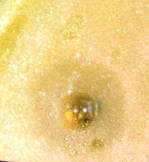

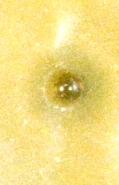


800 400 200 100 50

**g) NF7 - SCNE**

800 400 200 100 50

**SCNN**

800 400 200 100 50

**h) NF8 - SNNE**

800 400 200 100 50

**SNNN**

1. 400 200 100 50

**Figure. S6.** Anticandida activity of nanoformulations (NF) along with their non-nano derivatives (NN) showing zone of inhibition at each test concentration in µgmL^-1^.

**Table S1a.** Stability of prepared nanoformulations stored at 4°C

| **S.No.** | **Nano-formulation** | **Zero day** | | **After 3 months** | | **After 9 months** | | **After 12 months** | |
| --- | --- | --- | --- | --- | --- | --- | --- | --- | --- |
|  |  | **Z average (nm)** | **DI** | **Z average (nm)** | **DI** | **Z average (nm)** | **DI** | **Z average (nm)** | **DI** |
| 1 | NF1 | 51 | 0.324 | 53 | 0.335 | 55 | 0.254 | 51 | 0.243 |
| 2 | NF2 | 70 | 0.337 | 72 | 0.399 | 82 | 0.443 | 75 | 0.343 |
| 3 | NF3 | 47 | 0.284 | 48 | 0.291 | 51 | 0.320 | 45 | 0.150 |
| 4 | NF4 | 54 | 0.226 | 50 | 0.232 | 57 | 0.236 | 53 | 0.145 |
| 5 | NF5 | 76 | 0.194 | 81 | 0.272 | 86 | 0.403 | 73 | 0.435 |
| 6 | NF6 | 138 | 0.288 | 102 | 0.391 | 83 | 0.391 | 72 | 0.391 |
| 7 | NF7 | 42 | 0.339 | 49 | 0.336 | 58 | 0.274 | 50 | 0.313 |
| 8 | NF8 | 62 | 0.394 | 66 | 0.272 | 71 | 0.424 | 67 | 0.217 |

*****DI – Dispersity Index

**Table S1b.** Stability of prepared nanoformulations stored at 25°C

| **S.No.** | **Nano-formulation** | **Zero day** | | **After 3 months** | | **After 9 months** | | **After 12 months** | |
| --- | --- | --- | --- | --- | --- | --- | --- | --- | --- |
|  |  | **Z average (nm)** | **DI** | **Z average (nm)** | **DI** | **Z average (nm)** | **DI** | **Z average (nm)** | **DI** |
| 1 | NF1 | 51 | 0.324 | 63 | 0.396 | 61 | 0.397 | 102 | 0.255 |
| 2 | NF2 | 70 | 0.337 | 76 | 0.425 | 75 | 0.405 | 25 | 0.245 |
| 3 | NF3 | 47 | 0.284 | 58 | 0.384 | 58 | 0.384 | 109 | 0.111 |
| 4 | NF4 | 54 | 0.226 | 61 | 0.397 | 62 | 0.383 | 278 | 0.225 |
| 5 | NF5 | 76 | 0.194 | 85 | 0.221 | 85 | 0.221 | 54 | 0.101 |
| 6 | NF6 | 138 | 0.288 | 135 | 0.501 | 141 | 0.476 | 54 | 0.200 |
| 7 | NF7 | 42 | 0.339 | 60 | 0.365 | 60 | 0.365 | 30 | 0.243 |
| 8 | NF8 | 62 | 0.394 | 62 | 0.383 | 63 | 0.396 | 75 | 0.471 |

*****DI –Dispersity Index

**Table S2.** Net change in OD (average of 3 readings) at 570nm after 18hrs of exposure to nanoformulations on *Staphylococcus aureus*

| Concentration (µgmL^-1^) | 800 | 400 | 200 | 100 | 50 |
| --- | --- | --- | --- | --- | --- |
| Compounds |  |  |  |  |  |
| Tetracycline | -0.051 | -0.037 | -0.046 | 0.021 | 0.045 |
| Blank nano-formulation | 0.016 | 0.057 | 0.119 | 0.161 | 0.181 |
| 1 | 0.097 | 0.156 | 0.334 | 0.343 | 0.395 |
| NF1 | 0.018 | 0.025 | 0.032 | 0.036 | 0.041 |
| 2 | 0.252 | 0.255 | 0.256 | 0.260 | 0.275 |
| NF2 | 0.002 | 0.019 | 0.023 | 0.035 | 0.037 |
| 3 | 0.127 | 0.142 | 0.159 | 0.179 | 0.213 |
| NF3 | 0..031 | 0.034 | 0.042 | 0.053 | 0.062 |
| 4 | 0.092 | 0.145 | 0.147 | 0.153 | 0.165 |
| NF4 | 0.002 | 0.006 | 0.017 | 0.038 | 0.044 |
| 5 | 0.133 | 0.145 | 0.149 | 0.153 | 0.156 |
| NF5 | 0.021 | 0.023 | 0.032 | 0.042 | 0.045 |
| 6 | 0.104 | 0.137 | 0.161 | 0.205 | 0.294 |
| NF6 | 0.008 | 0.013 | 0.021 | 0.023 | 0.027 |
| 7 | 0.225 | 0.238 | 0.285 | 0.290 | 0.98 |
| NF7 | 0.004 | 0.006 | 0.017 | 0.020 | 0.033 |
| 8 | 0.187 | 0.197 | 0.213 | 0.218 | 0.221 |
| NF8 | 0.022 | 0.035 | 0.039 | 0.044 | 0.049 |
| Control | 0.967 | | | | |

^*^NF-Nanoformulation

**Table S3.** Net change in OD (average of 3 readings) at 570nm after 18hrs of exposure to nanoformulations on *Bacillus subtilis*

| **Concentration (**µgmL^-1^**)** | **800** | **400** | **200** | **100** | **50** |
| --- | --- | --- | --- | --- | --- |
| **Compounds** |  |  |  |  |  |
| **Tetracycline** | -0.024 | -0.011 | -0.004 | 0.01 | 0.015 |
| **Blank nano-formulation** | 0.026 | 0.065 | 0.173 | 0.253 | s0.266 |
| **1** | -0.008 | -0.031 | 0.015 | 0.016 | 0.038 |
| **NF1** | -0.003 | -0.004 | -0.01 | -0.01 | 0.002 |
| **2** | 0.078 | 0.105 | 0.189 | 0.198 | 0.202 |
| **NF2** | -0.021 | 0.012 | 0.254 | 0.388 | 0.473 |
| **3** | 0.008 | 0.102 | 0.192 | 0.203 | 0.206 |
| **NF3** | -0.036 | 0.02 | 0.116 | 0.231 | 0.234 |
| **4** | 0.104 | 0.108 | 0.135 | 0.136 | 0.144 |
| **NF4** | -0.006 | 0.005 | 0.141 | 0.144 | 0.409 |
| **5** | 0.063 | 0.108 | 0.117 | 0.134 | 0.136 |
| **NF5** | -0.004 | -0.017 | 0.055 | 0.159 | 0.3 |
| **6** | 0.011 | 0.013 | 0.023 | 0.033 | 0.086 |
| **NF6** | -0.007 | -0.004 | 0.001 | 0.004 | 0.006 |
| **7** | 0.027 | 0.029 | 0.031 | 0.034 | 0.035 |
| **NF7** | -0.028 | 0.007 | 0.009 | 0.012 | 0.014 |
| **8** | 0.017 | 0.018 | 0.022 | 0.024 | 0.026 |
| **NF8** | -0.01 | -0.018 | 0.008 | 0.015 | 0.02 |
| **Control** | 0.561 | | | | |

^*^NF-Nanoformulation

**Table S4.** Net change in OD (average of 3 readings) at 570nm after 18hrs of exposure to nanoformulations on *Pseudomonas aeruginosa*

| **Concentration (**µgmL^-1^**)** | **800** | **400** | **200** | **100** | **50** |
| --- | --- | --- | --- | --- | --- |
| **Compounds** |  |  |  |  |  |
| **Tetracycline** | -0.002 | -0.004 | -0.006 | 0.001 | 0.003 |
| **Blank nano-formulation** | 1.266 | 0.852 | 0.425 | 0.225 | 0.125 |
| **1** | 0.035 | 0.143 | 0.148 | 0.183 | 0.195 |
| **NF1** | -0.096 | -0.375 | -0.294 | -0.219 | -0.141 |
| **2** | 0.093 | 0.112 | 0.148 | 0.209 | 0.234 |
| **NF2** | -0.012 | -0.098 | -0.372 | -0.288 | 0.15 |
| **3** | 0.112 | 0.169 | 0.183 | 0.199 | 0.222 |
| **NF3** | -0.04 | -0.234 | -0.182 | -0.139 | 0.004 |
| **4** | 0.017 | 0.101 | 0.106 | 0.122 | 0.136 |
| **NF4** | -0.005 | -0.004 | -0.203 | -0.232 | 0.108 |
| **5** | 0.078 | 0.109 | 0.112 | 0.118 | 0.127 |
| **NF5** | -0.02 | -0.599 | -0.592 | 0.01 | 0.127 |
| **6** | 0.109 | 0.167 | 0.176 | 0.201 | 0.205 |
| **NF6** | -0.046 | -0.131 | -0.107 | -0.105 | 0.005 |
| **7** | 0.035 | 0.112 | 0.126 | 0.129 | 0.187 |
| **NF7** | -0.032 | -0.492 | -0.218 | -0.146 | -0.118 |
| **8** | 0.028 | 0.037 | 0.095 | 0.173 | 0.192 |
| **NF8** | -0.05 | -0.234 | -0.186 | -0.181 | 0.153 |
| **Control** | 1.022 | | | | |

^*^NF-Nanoformulation

**Table S5.** Net change in OD (average of 3 readings) at 570nm after 18hrs of exposure to nanoformulations on *Acinetobacter baumannii*

| **Concentration (**µgmL^-1^**)** | **800** | **400** | **200** | **100** | **50** |
| --- | --- | --- | --- | --- | --- |
| **Compounds** |  |  |  |  |  |
| **Tetracycline** |  |  |  |  |  |
| **Blank nano-formulation** | 0.027 | 0.065 | 0.125 | 0.223 | 0.257 |
| **1** | 0.004 | 0.009 | 0.012 | 0.029 | 0.043 |
| **NF1** | -0.029 | -0.027 | 0.003 | 0.003 | 0.013 |
| **2** | 0.104 | 0.106 | 0.109 | 0.119 | 0.123 |
| **NF2** | -0.009 | -0.005 | 0.049 | 0.071 | 0.073 |
| **3** | 0.039 | 0.049 | 0.077 | 0.099 | 0.128 |
| **NF3** | -0.031 | 0 | 0.021 | 0.039 | 0.047 |
| **4** | 0.41 | 0.458 | 0.511 | 0.515 | 0.517 |
| **NF4** | -0.01 | -0.007 | 0.007 | 0.275 | 0.363 |
| **5** | 0.123 | 0.151 | 0.216 | 0.219 | 0.298 |
| **NF5** | -0.015 | -0.005 | 0.209 | 0.296 | 0.364 |
| **6** | -0.003 | -0.002 | -0.001 | 0.016 | 0.025 |
| **NF6** | -0.037 | -0.005 | -0.001 | 0.006 | 0.02 |
| **7** | 0.045 | 0.048 | 0.069 | 0.076 | 0.143 |
| **NF7** | -0.038 | -0.007 | -0.006 | 0.062 | 0.070 |
| **8** | 0.035 | 0.037 | 0.040 | 0.042 | 0.058 |
| **NF8** | -0.035 | -0.013 | 0.054 | 0.077 | 0.082 |
| **Control** | 1.081 | | | | |

^*^NF-Nanoformulation
